# Urinary tract infections in children from the Gulf Cooperation Council countries: a literature review (2011–2022)

**DOI:** 10.3389/fped.2023.1163103

**Published:** 2023-07-17

**Authors:** May Albarrak, Mona Al Dabbagh, Hilal Al Hashami, Omar Alzomor, Ghassan Ghatasheh, Nervana Habashy, Ashraf Hassanien, Andrés Pérez-López

**Affiliations:** ^1^ Pediatric Infectious Diseases Department, Prince Sultan Military Medical City, Riyadh, Saudi Arabia; ^2^ Department of Pediatrics, Division of Infectious Diseases, King Abdulaziz Medical City, King Abdullah International Medical Research Center, King Saud Bin Abdulaziz University for Health Sciences, Jeddah, Saudi Arabia; ^3^ Pediatric Infectious Diseases Department, Lean Healthcare Certification, Royal Hospital, Muscat, Oman; ^4^ Pediatric Infectious Diseases Department, King Saud Medical City, Riyadh, Saudi Arabia; ^5^ Department of Pediatrics, Tawam Hospital, Al Ain, United Arab Emirates; ^6^ Pfizer, Dubai, United Arab Emirates; ^7^ Pfizer, Jeddah, Saudi Arabia; ^8^ Division of Microbiology, Sidra Medicine, Doha, Qatar; ^9^ Weill Cornell Medicine in Qatar, Doha, Qatar

**Keywords:** urinary tract infection, pediatric, Gulf Cooperation Council (GCC), antimicrobial susceptibility, antimicrobial resistance

## Abstract

Urinary tract infections (UTIs) are common healthcare-associated and community-acquired bacterial infections in children. Data on pediatric UTIs in the Gulf Cooperation Council (GCC) region (Bahrain, Kuwait, Oman, Qatar, Saudi Arabia, and the United Arab Emirates) have not been collated. Our aim is to review the published literature on the risk factors, etiology, antimicrobial susceptibility, and treatment of pediatric (aged <18 years) UTIs from healthcare and community settings in the GCC countries.

## 1. Introduction

Urinary tract infections (UTIs) are common in children (1–3). Up to 11% of children have had a UTI by 16 years of age, with higher infection rates in girls than boys (4–7). The diagnosis, prevalence and risk factors for UTIs may be stratified by patient sex or age, or the presence of underlying anatomical anomalies [such as vesicoureteral reflux (VUR)] that can lead to the recurrence of infection (1, 8). Awareness of the current risk factors for pediatric UTIs and factors contributing to recurrent UTIs can improve the clinical outcome of children with UTIs.

Uropathogenic *Escherichia coli* accounts for 80%–90% of pediatric UTIs (9). Resistance to common antibiotics used to treat UTIs, such as ampicillin and trimethoprim-sulfamethoxazole, is high among *E. coli* urinary isolates from children in the Gulf Cooperation Council (GCC) region [Bahrain, Kuwait, Oman, Qatar, Saudi Arabia, and the United Arab Emirates (UAE)] (10). Furthermore, isolates producing extended-spectrum β-lactamases (ESBLs) exhibit not only resistance to third- and fourth-generation cephalosporins and monobactams but also cross-resistance to other antibiotic classes, such as aminoglycosides and fluoroquinolones, are increasingly collected in GCC countries (10).

The challenges of managing pediatric UTIs in the GCC region have not been documented in a single article. Therefore, this review will collate data on risk factors, pathogens, resistance phenotypes, and antimicrobial management practices for pediatric UTIs within the region. Our objective is to provide a comprehensive assessment of the current knowledge on pediatric UTIs to improve diagnostic accuracy and help clinicians make rational therapeutic choices for UTIs, to improve the health of children in the region.

## 2. Methodology

### 2.1. Search strategy and selection criteria

This review evaluated relevant studies published between 1st January 2011 and 31st March 2022 on the PubMed and Google Scholar databases. We excluded non-English language articles, case reports, letters, books, editorials, notes, and conference abstracts.

The primary search terms were “urinary tract infection” and “pediatric”, followed by secondary terms relating to the review topics (shown in Supplementary Table S1). A free text search in title and abstract fields was also performed. Study methodology information and relevant study data were extracted from each article by two independent reviewers. Study information and findings not presented in-text are presented in Supplementary Tables S2–S4. Study data that were omitted from Tables 1, 2 or Supplementary Tables S2–S4 were not available in that publication.

**Table 1:** Summary table of study findings on pediatric UTIs in the GCC countries (2011–2022).

Study reference/country	Type of UTI	Patients (*N*)	*E. coli*, *Klebsiella* spp., ESBL-producers, *n*/*N* (%)	Study findings
Al-Saif et al. (11)/Saudi Arabia	CA	249 (≤14 y)	*E. coli*, 175/258 (67.8) ESBL-*E. coli*, 7/175 (4.0) *Klebsiella*, 32/258 (12.4) ESBL-*Klebsiella*, 3/32 (9.4)	Age (mo) at presentation of CA-UTI in boys (*n* = 62) was significantly earlier than in girls (*n* = 187) (6; IQR: 2–15 versus 45; IQR: 10–83, *P* < 0.001). Five of the ESBL isolates were from children less than one year old. *E. coli* and *Klebsiella* spp. constituted 64% (95% CI: 51–75) in boys versus 85% (95% CI: 79–90) of uropathogens in girls (*P* < 0.001). Susceptibility among uropathogens was highest to CXM, NIT, CTX, and GEN. Of the 10 (4%) ESBL-positive isolates *(E. coli*, 7; *Klebsiella*; 3), 5 were from children <1 y old. There were more ESBL isolates in the 2007–2009 than 2003–2006 period (8/124 versus 2/136; *P* = 0.05). All ESBL-positive isolates were susceptible to IPM.
Al-Otaibi and Bukhari (12)/Saudi Arabia	Hospitalized pts and out-patients; ESBL versus non-ESBL (adult and pediatric)	339 (including 233 pediatric, <18 y)	non-ESBL-*E. coli*, 176/233 (75.5) ESBL-*E. coli*, 57/233 (24.5)	More pediatric pts presented with non-ESBL-*E. coli* (75.5%) than with ESBL-*E. coli* (24.5%). Non-ESBL-*E. coli* UTI was more commonly found in children than adults (*P* < 0.0001). Compared with non-ESBL *E. coli*, ESBL-*E. coli* was more predominant in female pts (56%) compared to male pts (43%) (*P* < 0.0001). ESBL-*E. coli* UTIs were found more frequently in pts who had an underlying renal disease (*P* = 0.017), and those who underwent renal transplant (*P* = 0.017), children with VUR (*P* = 0.044), pts with post-surgical interventions (*P* < 0.0001), pts with recurrent UTI (*P* < 0.004), and in-patients (*P* < 0.0001). There were more deaths in the ESBL *E. coli* than the non-ESBL UTI group (*P* < 0.0001). ESBL-*E. coli* were often resistant to 3GCs, GEN and CIP (*P* < 0.0001). Non-ESBL-*E. coli* were resistant to antibiotics usually recommended for the initial therapy of UTIs including AMP and SXT (*P* < 0.0001).
Garout et al. (13)/Saudi Arabia	Single-/first episode (54.9%) and recurrent (45.1%)	153 (<5 y)	*E. coli*, 63/153 (41.2) *K. pneumoniae*, 30/153 (19.6) Similar in first versus recurrent UTI	*E. coli* as causative organism was 54.4% in boys versus 30.6% in girls (*P* = 0.003). The distribution of *E. coli* (49%), and non-*E. coli* (51%) in pts with normal US was comparable (*P* = 0.006), whereas for abnormal US, non-*E. coli* spp. were more common (73.6%) than *E. coli* (26.4%). Renal anomalies were found in 50.7% of those with recurrent episodes of UTI, whereas among those with first episode UTI, 10% had renal anomalies (*P* < 0.005).
Hendaus et al. (14)/Qatar	UTI among patients admitted to hospital with acute bronchiolitis	483 (data analysis group, 0–24 m)	NA	The overall prevalence of UTI in all groups was 10% (48/483) of cases (95% CI: 7.6%–12.9%). A significantly higher prevalence of UTI was observed in girls compared with boys (13.5% [95% CI: 9.5%–18.8%] versus 7.4% [95% CI: 5.1%–10.9%]; *P *= 0.023). Age at diagnosis >2 mo was significantly associated with a higher prevalence of UTI compared with age ≤2 mo (14.1% [95% CI: 10.2%–19.1%] versus 6.4% [95% CI: 4.1%–10.1%]; *P *= 0.005). In logistic regression analysis, being female (OR, 1.94 [95% CI: 1.09–3.49]); *P* = 0.025), older age at diagnosis (OR, 2.41 [95% CI: 1.29–4.51]; *P* = 0.006) were significantly associated with an increased risk for UTI. In multivariable logistic regression analysis, older age at diagnosis (adjusted OR, 3.65 [95% CI: 1.58–8.42]; *P* = 0.002) remained significantly associated with UTI.
Husain et al. (15)/Kuwait	First-episode	149 (≤12 y)	*E. coli*, 103/149 (69.1) *K. pneumoniae*, 18/149 (12.1)	Male children aged 0–6 mo accounted for 31%, of which 69 (87%) were not circumcised (*P *< 0.001). Prolonged hospitalization was associated with age <3 mo (*P *= 0.02) and duration of fever of more than 36 h (*P *= 0.001) but no association was found with prolonged hospitalization and the isolated organism. A low rate of VUR (7.5%) was found among children with first UTI and normal RUS; thus, it is safe to adopt new practice guidelines of no more MCUG imaging to detect VUR in this group of children. The resistance rates to CTX (empiric antibiotic for UTI at hospital) were 28% for *E. coli* and 6% for *Klebsiella*. No isolates were resistant to MEM and 0.8% were AMK-R.
Sharef et al. (16)/Oman	First-episode	175 (≤14 y)	*E. coli*, 120/175 (68.6) ESBL-*E. coli* 3/120 (2.5) *K. pneumoniae*, 30/175 (17.1) ESBL-*K. pneumoniae* 2/30 (6.6)	Overall, 73% of females and 27% of males had UTIs (*P* < 0.001). Significantly more male infants than females age <1 y had UTIs [67% and 33%, respectively (*P* < 0.001)]; however, the percentage of females were significantly higher in each of the older age groups (*P* < 0.001). While we observed a higher resistance to oral SXT in the uropathogens found in our study compared to other reports, resistance to IV antibiotics was much less. We recommend that the use of AMP, AMC and SXT is not justified as a first line of treatment, specifically in pts with acute pyelonephritis.
Kabbani et al. (17)/Saudi Arabia	Catheter-associated after cardiac surgery	413 (<14 y)	*E. coli*, 6/29 (20.7) ESBL-*E. coli*, 3/6 (50.0) *K. pneumoniae*, 7/29 (24.1) ESBL-*K. pneumoniae*, 3/7 (42.9)	Catheter-associated UTI incidence was 7%. We concluded that an increased duration of the urinary catheter (*P* < 0.001), the presence of congenital abnormalities of the kidney and urinary tract (*P* = 0.004), and the presence of syndromes [Down, DiGeorge and Noonan (*P* = 0.02)] comprised the main risk factors for catheter-associated UTI. Gram-negative organisms were the main causes for catheter-associated UTI, and one-third of them found to be resistant in this single-center study.
Alanazi (18)/Saudi Arabia	ED setting, CA); *E. coli* UTI (adult and pediatric)	101 with *E. coli*, including 31 pediatric (≤12 y)	*E. coli*, 29/31 (93.5) ESBL-*E. coli*, 1/29 (3.4)	High resistance was observed to AMP (74.2%) and SXT (51.7%) which commonly used as empirical treatments for UTIS, limiting their clinical use. The profile of 15/29 (51.7%) *E. coli* was resistance to both AMP and SXT while retaining susceptibility to CIP. No isolates were resistant to NIT.
Alanazi et al. (19)/Saudi Arabia	CA (adult and pediatric)	1,449 (including 269 pediatric, < 14 y)	NA	Among pediatric pts, most cases were single episode UTI (238/269, 88.5%), 173 (64.0%) were female; 238 (88.5%) received 1 antibiotic, 28 (10.4%) received 2 and 3 (1.1%) received ≥3. Broad-spectrum antibiotics were prescribed to 203 (75.5%); cephalosporins and penicillin were prescribed to similar proportions (∼44%) of pts. Among all pediatric pts, the rate of inappropriate antibiotic was 51.3%; 50.8% had been prescribed 1 antibiotic while 60.7% had been prescribed 2; 66.7% were prescribed cephalosporins, followed by penicillins (33.6%). The most frequent error was in dose level (44.2%), followed by dose duration (6.3%), dose frequency (5.9%), and dose selection (3.3%).
Hisham et al. (20)/Saudi Arabia	First-episode and recurrent	63	NA	No response to antibiotics within 48 h was noted for 48 (76.3%) cases. Resistance to PEN was 12.7%; 30.2% were multidrug-resistant; and 52.4% showed no drug resistance. No association (P > 0.1) between type of drug resistance (PEN-R, quinolone R, SXT-R, multidrug-resistant) and any of the following: age, sex, congenital anomalies, frequency of UTI, atypical features of UTI, hydronephrosis of UTI, degree of reflux.
Alfakeekh et al. (21)/Saudi Arabia	Hospitalized pts with primary childhood nephrotic syndrome	111 (including 21 with UTI) (≤14 y)	NA	Higher annual cumulative dose of steroids, and combination of primary and secondary immunosuppressants were the highest independent risk factors for infection. About half of infection encountered by primary childhood nephrotic syndrome pts were upper respiratory tract infection (52%) followed by UTI (25%) and pneumonia (20%).
Awean et al. (22)/Qatar	ED, out-patient and in-patient settings	254 (ESBL bacteria, 68; non-ESBL, 186)	ESBL-*E. coli*, 55/170 (32.4) non-ESBL-*E. coli*, 115/170 (67.6) ESBL-*Klebsiella* spp., 8/25 (32.0) non-ESBL- *Klebsiella* spp. 17/25 (68.0)	There are no statistically significant risk factors predisposing to infection with ESBL- or non-ESBL-bacteria with regards to patient age, previous use of antibiotics, recurrent UTI and underlying renal anomalies, (*P* > 0.05), and no significant statistical difference between the two groups and an abnormal renal US or abnormal VCUG (*P* > 0.05).
Hameed et al. (23)/Saudi Arabia	First-episode, CA	202 (0–14 y)	*E. coli*, 153/202 (75.7) *K. pneumoniae*, 19/202 (9.4)	Significantly higher proportion of female than male pts (19.8% versus 80.2%). Among female pts, uropathogen was *E. coli* in 82.1% and non-*E. coli* in 17.9%. Distribution of *E. coli* (*n* = 20) and non-*E. coli* (*n* = 20) was equal among male pts. All isolates were susceptible to carbapenems. Over half of *E. coli* isolates were resistant to 2 or more antibiotics. AMC and SXT are not appropriate choices for empiric therapy for CA-UTI, whereas 3GCs remain a good choice.
Mohammed et al. (24)/Bahrain	First-episode and recurrent	125 (<1 y)	*E. coli*, 69/125 (55.2) ESBL-*E. coli*, 30/69 (43.4) *K. pneumoniae*, 44/125 (35.2) ESBL-*K. pneumoniae*, 14/44 (31.8)	68% male and 32% female pts. Recurrent UTI in 12%. Fever was the most common clinical presentation (74, 59.2%), followed by neonatal jaundice (46, 36.8%). The most common pathogen was *E. coli* (55.2%), followed by *K. pneumoniae* (35.2%), with high rates of ESBL-producers (43.4% of the total *E. coli* and 31.8% of *K. pneumoniae*). Approximately one-third of infants with UTI had urological anomalies.
Abuzeyad et al. (25)/Bahrain	ED setting	104 (>1 mo–14 y)	*E. coli*, 67/104 (64.4) ESBL-*E. coli*, 31/67 (46.3) *K. pneumoniae*, 4/104 (3.8)	Ratio of female to male pts was 5:1. Fever (64.4%), was the most common symptom followed by vomiting (43.3%), and abdominal pain (28.8%). *E. coli* was the most common uropathogen (36, 34.6% cases; 31 ESBL-positive). Among the empiric antibiotics used, CXM was more active than AMC against *E. coli*.
Safdar et al. (26)/Saudi Arabia	NA	315 (≤14 y)	*E. coli*, 38/115 (33.0) ESBL-*E. coli*, 8/38 (26.7) *K. pneumoniae*, 25/115 (21.7) ESBL-*K. pneumoniae*, 5/25 (20.0)	Prevalence of UTI was 36.8%, with a higher rate among female than male pts (42.1% versus 32.0%). Pyuria was associated with a positive urine culture (*P* = 0.001). VUR was found in 13% of pts diagnosed with UTI, compared with 2% without UTI (*P* < 0.001). Non-*E. coli* cases were more likely to have VUR, compared with *E. coli* (*P* = 0.0406). Pyuria was more sensitive to detect *E. coli* UTI, compared with non-*E. coli* (83% versus 65%, *P* = 0.044). Nitrite appears also to be significantly more sensitive in detecting *E. coli* UTI than non-*E. coli* UTI (20% versus 7%, *P* = 0.046).
Alavudeen et al. (27)/Saudi Arabia	In-patients	132 (<10 y)	*E. coli*, 42/132 (31.8) *K. pneumoniae*, 33/132 (25.0)	UTI was more prevalent among males than females in the younger age groups (≤48 mo, 63.9% versus 36.1%; > 48 mo, 25% versus 75%). Fever was the main symptom (84.1%), followed by diarrhea (18.2%). *E. coli* was the most common uropathogen (31.8%), followed by *K. pneumoniae* (25%). Cephalosporins were the most commonly prescribed drug class; the most prescribed agents were CRO, VAN, MEM and CXM. All *E. coli* and *K. pneumoniae* were AMP-R.
Al Nafeesah et al. (28)/Saudi Arabia	CA	202 (0–14 y)	*E. coli*, 153/202 (75.7) non-*E. coli*, 49/202 (24.3) most commonly, *K. pneumoniae*	Female pts accounted for 80.2% of the study population. There was also a significant association with prior use of an antibiotic and previous history of UTI (*P* = 0.011 and 0.012, respectively). For non-*E. coli* UTIs, abnormalities in renal US and VCUG were seen in 66.7% and 76.7% of pts, respectively, both significantly higher than in *E. coli* UTIs (*P* = 0.008 and *P* = 0.01, respectively). A higher frequency of children with *E. coli* UTIs had a length of stay of ≤1 wk than that of non-*E. coli* UTIs (*P *= 0.042).
Alrasheedy et al. (29)/Saudi Arabia	CA, including severe/complicated	280 (1–10 y)	NA	The prevalence of UTI was 25.8%. This trial showed that the prevalence of UTI varied widely by age, gender and regional place and comorbidities. UTI was more common among females. The main presenting complaint was urethritis. Nearly a sixth of children developed severe UTI.
Alzahrani et al. (30)/Saudi Arabia	CA and HA	118	*E. coli*, 52/118 (44.1) ESBL-*E. coli*, 14/52 (26.9) *K. pneumoniae*, 12/118 (10.2) ESBL-*K. pneumoniae*, 1/12 (8.3)	NIT (19%) was most commonly prescribed to in-patients and out-patients, followed by SXT (16%), AMC (15%), CXM (10%), azithromycin (8%), CRO (7%), and CIP (4%). AMK, AMX, AMP, FEP, IPM, phenoxymethylpenicillin were prescribed less commonly due to high resistance.
Edun et al. (31)/Saudi Arabia	Concurrent UTI in bronchiolitis	407 (0–2 y)	NA	270/407 (66%) of pts with bronchiolitis were tested for UTI: only 3/270 (1.1%) were positive for urine culture and urinalysis. This gives the prevalence of UTI to be <1% based on the total number of diagnosed cases (*N* = 407).
El-Naggari et al. (32)/Oman	First-episode and recurrent	405 (<14 y)	*E. coli*, 307/405 (75.8) *K. pneumoniae*, 42/405 (10.4)	In first UTIs (*n* = 175), *E. coli* was the leading pathogen (69%), then *K. pneumoniae* (17%), and ESBL-producers (3%). In recurrent UTI (*n* = 230), the most common isolated pathogen was *E. coli* 187 (81.3%), then *K. pneumoniae* 12 (5.1%), ESBL-producers (7%). There were significant differences between first and recurrent UTI pts in incidence of *E. coli* (*P* = 0.001), *K. pneumoniae* (*P* < 0.001) and ESBL-producers (*P* = 0.042). Higher AMC and cephalosporin resistance was found in recurrent than first UTI. The authors recommend CIP and NIT for empiric treatment of first and recurrent UTI.
Saeed et al. (33)/Bahrain	*E. coli* (adult and pediatric)	3,044 (including 604 pediatric: 248 aged <1 y; 356 aged 1–15 y)	*E. coli*, 604/3,044 (19.8) ESBL-*E. coli*, 216/604 (35.8)	ESBL producers were 107/248 (43.1%) in the <1 y group and 109/356 (30.6%) in the 1–15 y group. The distribution of *E. coli* overall and ESBL-producers was lower among the pediatric pts (<12% and <10%, respectively) than adults (>30%). Among ESBL producers, CIP susceptibility was higher in the <1 y group than other age groups; yet, NIT and FOF susceptibility was lowest in this group.
Shaaban et al. (34)/Bahrain	NA	242 (<14 y)	*E. coli*, 166/242 (68.6) *K. pneumoniae*, 25/242 (10.3) ESBL-*E. coli*, 39/166 (23.5)	53 male (21.9%) and 189 female (78.1%) pts (*P* < 0.01); categorized into 3 age groups: 4 (1.7%) neonates (<28 d), 51 (21.1%) infants (28 d–1 y) and 186 (77.2%) were children (1–14 y) (*P* < 0.01). The rate of *E. coli* was higher in females than males (77.8% versus 35.8%) pts (*P* < 0.01), and highest among neonates (75%), followed by children (69.5%) and infants (64.7%) (*P* < 0.001). The frequency of *K. pneumoniae* was highest in infants (25%) followed by neonates (15.7%) followed by children (8.6%) (*P* < 0.001). The rate of ESBL-*E. coli* was significantly different between male (42.1%) and females (21.0%) pts (*P* < 0.01), and between age groups (36.4% for neonates, 0% for infants, and 20.7% for children) (*P* = 0.016). *E. coli* showed highest resistance to CFZ (94%, *P* < 0.01), followed by AMP (62.7%, *P* < 0.01) and CAZ (46.7%, *P* < 0.01), and highest susceptibility to NIT (99%) and AMK (98.4%). *E. coli* resistance to CIP and CXM increased significantly (9.5% to 23.3% [*P* = 0.015] and (3.6%to 38.6%) [*P* < 0.008], respectively) over the study period (2018–2020).
Safdar et al. (35)/Saudi Arabia	Febrile	73 (0–14 y)	NA	Urinary NGAL is a poor biomarker for the diagnosis of febrile UTI: No significant difference was observed between urinary NGAL levels in pts with and without UTIs (*P* = 0.17). There was a positive monotonic correlation between NGAL and CRP levels (*n* = 73, *P* < 0.001).
Eltai et al. (36)/Qatar	ESBL-producing	727 positive urine cultures; 635 (87.3%) were identified as *Enterobacteriaceae* among which 201/635 (31.7%) were ESBL-positive. (0–15 y)	ESBL-*E. coli* 166/201 (82.6) ESBL-*K. pneumoniae* 22/201 (10.9)	Among pts with ESBL-positive UTIs, 167 (83%) were female. 40.8% were aged 2–5 y and the remainder were evenly distributed between age groups (<2 and 6–15 y). 83% of all ESBL-producing bacteria were *E. coli*. All ESBL-producing isolates showed 100% resistance to AMP, and to all cephalosporins including CEF, CFZ, CRO and CEF. Low resistance was recorded to carbapenems (between 2.5% to MEM, ETP and 10% to IPM). Among the β-lactam/β-lactamase inhibitor combinations, 9% were TZP-R, whereas 99% were AMC-R. Regarding aminoglycosides, all the isolates were susceptible to AMK, and 24.4% were GEN-R. The resistance prevalence to other classes of antibiotics namely, cefoxitin, nitrofurantoin, trimethoprim/sulfamethoxazole and ciprofloxacin was 19.4, 13, 59.7 and 36%, respectively. CTX-M-G1 was the most frequent ESBL among *E. coli* (58/95, 61.1%) and the TEM, SHV and CTX-MG1 combination among *K. pneumoniae* (6/13, 46.2%). Significant associations (*P *< 0.05) were found between the presence of TEM and NIT and SXT resistance; between SHV and NIT resistance; and between CTXM-G2 and TZP resistance.
Ahmad et al. (37)/Saudi Arabia	Enterococcal	317 (newborns)	NA	30 (9.5%) enterococcal strains were isolated from 317 samples. Of these, 17 (56.6%) were from male and 13 (43.4%) were from female pts. Isolates were resistant to clindamycin (100%), cefoxitin (95%), and nalidixic acid (93.3%), but susceptible to CIP, IMP and AMC (resistance rates, 6.7%, 10% and 10.8%, respectively).
Al Mana et al. (38)/Qatar	Lower UTI, CRE	29 (≤15 y)	*E. coli*, 19/30 (63.3) *K. pneumoniae*, 9/30 (30.0)	The most common carbapenemases produced were OXA-48-like enzymes (46.6%) and NDM enzymes (40%). The most common OXA-48-like carbapenemase observed is OXA-244 (50% of OXA-48-like enzymes), followed by OXA-181 and OXA-48. Among the NDM producers, 66.7% (8/12) were from pts from the Indian subcontinent, compared with 14.3% among the OXA-48-like carbapenemase producers. All isolates showed antimicrobial resistance but were susceptible to colistin and tigecycline.

3GC, third-generation cephalosporin; AMC, amoxicillin-clavulanic acid (Augmentin); AMK, amikacin; AMP, ampicillin; AMX, amoxicillin; CA, community-acquired; CA-UTI, community-acquired urinary tract infection; CAZ, ceftazidime; CFZ, cefazolin; CIP, ciprofloxacin; CRE, carbapenem-resistant Enterobacterales; CRO, ceftriaxone; CRP, C-reactive protein; CTX, cefotaxime; CXM, cefuroxime; d, days; ESBL, extended-spectrum β-lactamase; FOF, fosfomycin; GEN, gentamicin; HA, hospital-acquired; IPM, imipenem; IQR, interquartile range; IV, intravenous; MCUG, micturating cystourethrogram; MEM, meropenem; mo, months; NA, data not available/reported; NGAL, neutrophil gelatinase-associated lipocalin; NIT, nitrofurantoin; OR, odds ratio; pts, patients; R, resistant; SXT, trimethoprim-sulfamethoxazole (Bactrim or co-trimoxazole); TZP, piperacillin-tazobactam (Tazocin); US, ultrasound; UTI, urinary tract infection; VCUG, voiding cystourethrogram; VUR, vesicoureteral reflux; wk, weeks; y, years.

**Table 2:** Antimicrobial susceptibility or resistance among *Escherichia coli*, *Klebsiella spp.*, and ESBL-producing isolates from pediatric UTIs in the GCC countries (2011–2022).

Organism/study reference/country	Total isolates (*N*)	S or R^a^	Penicillins (AMP)	β-lactam combinations (AMC or TZP)	Cephalosporins (CAZ, CEF, CFZ, CRO, CTX, CXM, FEP or FOX)	Carbapenems (IPM, MEM or ETP)	Aminoglycosides (AMK, GEN or TOB)	Fluoroquinolones (CIP, LVX or NOR)	SXT or TMP	NIT
*E. coli*										
Al-Saif et al. (11)/Saudi Arabia	175	S	27.4	TZP: 93.1	CAZ, CRO, CTX, CXM: 94. 8	IPM: 100 (167/167)	AMK: 98.0 (102/104) GEN: 90.8	NT	SXT: 44.0	96.0
Husain et al. (15)/Kuwait	103	R	55	AMC: 33 TZP: 8	CAZ: 22 CEF: 48 CTX: 28	MEM: 0	AMK: 0	NT	TMP: 38	9
Sharef et al. (16)/Oman	120 (including 3 ESBL-producing *E. coli*)	R	76	AMC: 24 TZP: 2	CRO, CTX: 18 CXM: 19	MEM: 1	AMK: 0 GEN: 11	CIP: 18	SXT: 50	2
Kabbani et al. (17)/Saudi Arabia	3	R	3/3	NT	CFZ: 1/3 CTX: 1/1	IPM: 0/1	GEN: 0/2	CIP: 0/3	SXT: 1/3	0/3
	3 ESBL-producing	R	1/1	NT	CTX: 1/1	IPM: 0/2 MEM: 0/3	AMK: 0/3 GEN: 3/3	CIP: 2/2	SXT: 3/3	0/3
Alanazi et al. (18)/Saudi Arabia	29	R	82.8	AMC: 10.3	CFZ: 13.8	NT	NT	CIP: 3.4	SXT: 51.7	0.0
	7 (age, <2 y)	R	6/7	AMC: 1/7	CFZ: 1/7	NT	NT	CIP: 0/7	SXT: 6/7	0/7
	12 (age, 2–6 y)	R	75.0	AMC: 16.7	CFZ: 16.7	NT	NT	CIP: 0.0	SXT: 50.0	0.0
	10 (age, 7–12 y)	R	80.0	AMC: 0.0	CFZ: 0.0	NT	NT	CIP: 10.0	SXT: 30.0	0.0
	2 (age, 13–17 y)	R	1/2	AMC: 0/2	CFZ: 0/2	NT	NT	CIP: 0/2	SXT: 1/2	0/2
Hameed et al. (23)^b^/Saudi Arabia	153	R	66.2	AMC: 29.6	CFZ: 22.3 CTX: 5.7	IPM, MEM: 0.0	9.6 (GEN)	CIP: 14.8	SXT: 57.1	2.2
Abyzeyad et al. (25)/Bahrain	36	S	NT	AMC: 52.8	CXM: 75.0	NT	NT	NT	NT	NT
	31 ESBL-producing	S	NT	AMC: 0.0	CXM: 0.0	NT	NT	NT	NT	NT
Alavudeen et al. (27)^c^/Saudi Arabia	42	S	0.0	AMC: 50.0 TZP: 92.9	CAZ, CRO: 92.9 CTX, FOX: 71.4 CXM: 42.9 FEP: 78.6	IPM: 78.6 MEM: 92.9 ETP: 92.9	AMK, GEN: 92.9	CIP: 71.4 LVX: 92.9	SXT: 78.6	42.9
Alzahrani et al. (30)^d^/Saudi Arabia	52	R	94.1 (32/34)	AMC: 57.1 (8/14) TZP: 14.3 (2/14)	CAZ: 0/4 CEF: 92.3 (12/13) CRO: 25.0 (3/12) CTX: 0/1 CXM: 46.7 (7/15) FEP: 2/7 FOX: 76.9 (10/13)	IPM: 6.5 (2/31) MEM: 1/9	AMK: 5.4 (2/37) GEN: 1/8	CIP: 31.8 (7/22) LVX: 0/2 NOR: 25.0 (5/20)	SXT: 55.6 (25/45)	4.3 (2/47)
	14 ESBL-producing	R	8/8	AMC: 2/6 TZP: 7.7 (1/13)	CAZ, CXM: 3/3 CEF, FOX: 2/2 CRO: 9/9 FEP: 100 (13/13)	IPM, MEM: 0.0 (0/11)	AMK: 7.7 (1/13) GEN: 63.6 (7/11)	CIP: 72.7 (8/11) LVX: 0/2 NOR: 0/1	SXT: 3/9	0.0 (0/13)
El-Naggari et al. (32)/Oman	120 (single-episode UTIs)	R	76	AMC: 24 TZP: 2	CRO, CTX: 18 CXM: 19	0	AMK: 0 GEN: 11	CIP: 18	SXT: 50	2
	187 (recurrent UTIs; including 16 ESBL-producing *E. coli*)	R	74	AMC: 37 TZP: 5	CRO, CTX: 30 CXM: 31	0	AMK: 3 GEN: 10	CIP: 13	SXT: 44	1
Saeed et al. (33)^e^/Bahrain	107 ESBL-producing (age, < 1 y)	S	NT	NT	NT	NT	NT	62.6	SXT: 46.7	71.0
	109 ESBL-producing (age, 1–15 y)	S	NT	NT	NT	NT	NT	CIP: 38.6 (27/70)	SXT: 21.4 (15/70)	100 (70/70)
Shaaban et al. (34)/Bahrain	166	R	62.7 (89/142)	AMC: 31.5 (41/130) TZP: 3.7 (5/136)	CAZ: 46.7 (42/90) CFZ: 94.0 (47/50) CRO: 37.1 (49/132) CXM: 33.8 (49/145)	NT	AMK: 1.6 (2/127) GEN: 8.7 (11/127)	CIP: 22/138 (15.9)	SXT: 36.8 (53/144)	1.0 (1/96)
	39 ESBL-producing	R	100 (36/36)	AMC: 86.2 (25/29) TZP: 9.7 (3/31)	CAZ, CRO: 97.0 (32/33) CFZ: 100 (29/29) CXM: 96.0 (24/25)	NT	AMK: 3.0 (1/33) GEN: 13.7 (4/29)	CIP: 25.8 (8/31)	SXT: 55.9 (19/34)	0.0 (0/23)
*Klebsiella* spp.										
Al-Saif et al. (11)/Saudi Arabia	32 *Klebsiella* spp.	S	0.0	TZP: 87.5	CAZ, CRO, CTX: 90.6 CXM: 87.5	IPM: 100	AMK: 95.0 (19/20) GEN: 90.6	NT	SXT: 75.0	71.8
Husain et al. (15)/Kuwait	17 *K. pneumoniae*	R	59	AMC: 29 TZP: 12	CAZ: 0 CEF, CTX: 6	MEM: 0	AMK: 6	NT	TMP: 18	35
Sharef et al. (16)/Oman	30 *Klebsiella* spp., including 2 ESBL-producing *K. pneumoniae*	R	100	AMC: 23 TZP: 10	CTX, CRO: 7 CXM: 17	MEM: 0	AMK: 0 GEN: 7	CIP: 10	SXT: 20	13
Kabbani et al. (17)/Saudi Arabia	4 *K. pneumoniae*	R	2/2	NT	CTX: 0/2	IPM: 0/2 MEM: 0/1	AMK: 0/1 GEN: 1/3	CIP: 0/4	SXT: 0/4	0/3
	3 ESBL-producing *K. pneumoniae*	R	1/1	NT	CFZ, CTX: 1/1	IPM: 0/1 MEM: 0/1	GEN: 0/3	CIP: 1/3	SXT: 0/2	0/3
Hameed et al. (23)/Saudi Arabia	19 *K. pneumoniae*	R	94.7	AMC: 33.3	CFZ: 100 CTX: 0.0	IPM: 0.0 MEM: 0.0	GEN: 0.0	CIP: 0.0	SXT: 50.0	66.7
Alavudeen et al. (27)^f^/Saudi Arabia	33 *K. pneumoniae*	S	0.0	AMC: 27.3 TZP: 45.5	CAZ, CRO, CXM, FEP: 18.2 CTX: 54.5 FOX: 45.5	IPM: 54.5 MEM: 100 ETP: 63.6	AMK: 72.7 GEN: 45.5	CIP: 36.4 LVX: 54.5	SXT: 63.6	45.5
Alzahrani et al. (30)^g^/Saudi Arabia	11 *K. pneumoniae*	R	9/9	AMC: 2/4 TZP: 2/5	CEF: 3/3 CRO: 2/6 CXM: 2/2 FOX: 1/1	IPM: 0/6 MEM: 1/5	AMK: 0/6 GEN: 0/1 TOB: 1/1	CIP: 0/3 LVX: 0/2 NOR: 1/5	SXT: 63.6	20.0
Shaaban et al. (34)/Bahrain	25 *K. pneumoniae*	R	95.2 (20/21)	AMC: 25.0 (5/20) TZP: 13.6 (3/22)	CAZ: 38.5 (5/14) CFZ: 83.3 (5/6) CRO: 22.7 (5/22) CXM: 28.6 (6/21)	NT	AMK: 4.8 (1/21) GEN: 10.0 (2/20)	CIP: 9.5 (2/21)	SXT: 27.2 (6/22)	7.1 (1/14)

Where an antimicrobial agent is not listed, study data on that agent were not available. Details of the testing methodologies and other agents not shown are presented in Supplementary Table S3.

AMC, amoxicillin-clavulanic acid (Augmentin or co-amoxiclav); AMK, amikacin; AMP, ampicillin; CAZ, ceftazidime; CEF, cephatholin; CFZ, cefazolin; CIP, ciprofloxacin; CRO, ceftriaxone; CTX, cefotaxime; CXM, cefuroxime; ESBL, extended-spectrum β-lactamase; ETP, ertapenem; FEP, cefepime; FOX, cefoxitin; GEN, gentamicin; IPM, imipenem; MEM, meropenem; NIT, nitrofurantoin; NOR, norfloxacin; NT, not tested; R, resistant; S, susceptible; spp., species; SXT, trimethoprim-sulfamethoxazole (Bactrim or co-trimoxazole); TGC, tigecycline; TMP, trimethoprim; TOB, tobramycin; TZP, piperacillin-tazobactam (Tazocin); and y, years.

^a^%S or %R are calculated to 1 decimal place, or as presented in the article where numerators are not given. %S or %R not presented when N < 10. When denominator is different to the total isolates (*N*), numerator, denominator and % are presented.

^b^ %R includes isolates that are in the intermediate category.

^c^ Reported agents also included fosfomycin (*E. coli*, %S: 14.3%) and tigecycline (*E. coli*, %S: 92.9%).

^d^ Reported agents also included aztreonam (*E. coli*, R isolates: 1/9; and ESBL-positive *E. coli*, %R: 100% [11/11]), colistin (*E. coli*, R isolates: 0/2), nalidixic acid (*E. coli*, R isolates: 1/3), and tigecycline (*E. coli*, R isolates: 0/2).

^e^ Reported agents also included fosfomycin (*E. coli* <1 y, %S: 78.5%; and *E. coli* 1–15 y, %S: 98.6% [69/70]).

^f^ Reported agents also included fosfomycin (*K. pneumoniae*, %S: 36.4%) and tigecycline (*K. pneumoniae*, %S: 81.8%).

^g^ Reported agents also included aztreonam (*K. pneumoniae*, R isolates: 0/3) and cedoxitine (*K. pneumoniae*, R isolates: 0/2).

### 2.2. Studies included in this review

Following initial identification and screening of publication titles, 38 full-text articles remained (Bahrain, 4; Kuwait, 1; Oman, 4; Qatar, 5; Saudi Arabia, 23; and the UAE, 1). Nine articles (Oman, 2; Qatar, 1; Saudi Arabia, 5; and the UAE, 1) were excluded for not differentiating data for extraction.

The final GCC study number totaled 28: 24 retrospective studies (11–34) and 4 cross-sectional studies (35–38) (Supplementary Table S4). One set of clinical guidelines (39) was included. Four studies included adult patient data (adult ages defined as >12 years (18), >15 years (19), >15 years (33) and >18 years (12):), presented separately from pediatric patients (Supplementary Table S4).

## 3. Diagnosis of pediatric UTIs in the GCC countries

### 3.1. Urine collection

The Saudi Pediatric Infectious Diseases Society (SPIDS) guidelines on community-acquired UTI (CA-UTI) recommend transurethral catheterization for collecting urine from infants and non-toilet-trained children to minimize bacterial contamination from the skin (39). Catheterization was used in 15 of all 28 studies (53.6%) (Supplementary Table S2) (11, 13, 14, 16, 17, 22, 24, 26–28, 30–32, 36, 38) and specified for younger children (≤2 years) in 5 studies (13, 26, 28, 36, 38). One study reported catheterization use for most patients aged <2 years and midstream collection for most aged >2 years (Supplementary Table S2) (13). In the community setting, clean catch samples may be preferred to catheterization (16). Clean catch urine was used in 13 of all studies (46.4%) (11, 13, 16, 18, 19, 22, 25–27, 30, 33, 35, 38) and suprapubic aspiration in 8 (28.6%) (17, 22, 24, 26, 28, 30, 32, 36). Urinary bag collection [not recommended by SPIDS (39)] was used in three studies (10.7%) (11, 30, 37) and excluded in three (10.7%) (13, 24, 28). Seven studies (25.0%) omitted the method of urine collection (12, 15, 20, 21, 23, 29, 34).

### 3.2. Urine testing

SPIDS defines UTI as significant bacteriuria in a symptomatic patient (febrile and/or with urinary symptoms) and recurrent UTI as ≥2 episodes of symptomatic UTI within 12 months (39). Their threshold for significant bacteriuria in colony-forming units (CFU) ranges from any number for suprapubic aspiration to ≥100,000 CFU/ml for clean catch urine. The American Association of Pediatrics (AAP) threshold is 50,000 CFU/ml for catheterization in febrile infants aged 2–24 months (40).

Positive pyuria by urine microscopy was an inclusion criterion of two studies (Supplementary Table S2) (15, 28) and was recorded in 25.6%–92.6% of patients (16, 23, 24, 26, 28). One study found an independent association between pyuria and positive urine culture (*P* = 0.001) (Table 1) (26). Pyuria (*P* = 0.044) and nitrite (*P* = 0.046) were significantly sensitive to detect *E. coli* UTI, compared with non-*E. coli* UTI (Table 1) (26).

### 3.3. Renal and bladder imaging

SPIDS advocates renal and bladder ultrasonography as a safe, noninvasive procedure for the detection of abnormalities and renal infections in preference to micturating or voiding cystourethrogram (MCUG or VCUG) (39). Renal ultrasound was performed for 66.4%–91.3% of patients, and abnormal findings were recorded in 11.4%–51.0% (Supplementary Table S4) (13, 15, 22, 24, 28). Overall, 11.5% (104/901) of patients across seven studies had VUR (Supplementary Table S4) (13, 15, 20, 22, 24, 26, 27). Following MCUG, VUR was detected in 13.0% of patients with a first UTI and 23.1% with recurrent UTI (Supplementary Table S4) (15). Among patients with ESBL-producing uropathogens, 42.9% had VUR (22). Children with their first UTI and normal renal ultrasound were less likely to have VUR and were recommended not to undergo MCUG (Table 1) (15).

### 3.4 Symptoms and biomarkers

The predominant symptom was fever (50.3%–86.6%), with a regional prevalence of 60.7% (784/1,292) (Supplementary Table S4) (15, 16, 23–25, 27, 32). Fever was more common in children aged ≤2 years (16, 24) in two studies, and first vs. recurrent UTI [50.3% and 24.8%, respectively (*P* < 0.001)] (Supplementary Table S4) (32). Two studies included only febrile children (14, 35).

Other common symptoms (>20% of each study cohort) were urinary frequency, urgency or dysuria, nausea, vomiting, and abdominal pain (Supplementary Table S4) (15, 16, 25). Nausea and/or vomiting rates were higher in patients aged ≤2 years (*P* = 0.002), whereas rates of abdominal pain and urinary symptoms were higher in older children [2–14 years (*P* < 0.001)] (16). In addition, nausea and/or vomiting and abdominal pain were significantly more common in first vs. recurrent UTI (*P* < 0.001) (Supplementary Table S4) (32).

Blood C-reactive protein (>5 mg/L) did not significantly change with patient age (16) or between recurring and first-episode UTI (32). SPIDS advises against routinely obtaining these laboratory parameters in children >3 months with suspected UTI who otherwise appear healthy (39).

## 4. Epidemiology of pediatric UTIs in the GCC countries

### 4.1. Prevalence of pediatric UTIs in the GCC countries

#### 4.1.1. Patient age

The prevalence of pediatric UTI across four studies including adults was 18.6%–68.7% (12, 18, 19, 33). In three studies, UTI was more common among younger patients aged <2 years (13, 15, 20), whereas two studies found a higher rate of UTI in patients aged 1–14 years (25, 34). Univariate and multivariate analyses showed that CA-UTI was associated with ages 1–6 years (29).

#### 4.1.2. Patient sex

Overall, 65.0% (2,550/3,924) of UTI patients were girls. More girls than boys had a UTI in 15 studies (Supplementary Table S4) (11, 12, 14, 19, 22, 23, 25, 26, 28–30, 32, 34, 36, 38); the difference was statistically significant in three studies (one including adults) (Supplementary Table S4) (12, 14, 34). Conversely, more boys had UTIs in seven studies (13, 15, 17, 20, 24, 27, 37), with younger patients (<5 years) (13, 24, 37) or higher rates of UTIs in younger males (<48 months) and older females (overall, 48 months to 14 years) [15 (not significant), 16 (*P* < 0.001), 27 (*P* = 0.001)]. Boys presented with CA-UTI at a significantly younger age than girls (Table 1) (11).

##### 4.1.2.1 Circumcision status

In other studies, being uncircumcised was associated with UTI (7, 41). In the current review, only three studies examined circumcision (13, 15, 23). In one study, most boys aged <6 months with a first UTI were uncircumcised (87%) (Table 1) (15). The circumcision rate was 67.5% among patients aged <14 years (23), whereas, in the other study, UTIs were more common in younger patients (<2 years), even in circumcised males (13).

### 4.2. Risk factors associated with pediatric UTIs in the GCC countries

#### 4.2.1. Patient-related risk factors for pediatric UTIs

Female sex (*P* = 0.018), antibiotic use (*P* = 0.015) and VUR (*P* = 0.019) were associated with UTI (26). Prolonged hospitalization (>7 days) was associated with age <3 months (*P* = 0.02) and duration of fever of >36 h (*P* = 0.001), but not with the isolated uropathogen (Table 1) (15).

Patient comorbidities (such as hydronephrosis and congenital abnormalities) were associated with UTI (*P* = 0.05) (29). UTI among patients with acute bronchiolitis was associated with female sex (*P* = 0.025) and age >2 months (*P* = 0.006) (Table 1) (14). In another study of bronchiolitis, the youngest patients (<3 months) were more likely to be tested for a UTI (*P* < 0.0001) (31).

Following cardiac surgery, 7.0% of patients had UTIs (all catheter-associated) (17). Prolonged urinary catheterization, the presence of congenital abnormalities of the kidney and urinary tract, and Down, DiGeorge, and Noonan syndromes were associated with UTI (Table 1) (17).

#### 4.2.2. Association of UTIs with *E. coli* vs. non-*E. coli* uropathogens

Compared with *E. coli*, non-*E. coli* UTIs were associated with hospitalization >7 days (*P* = 0.042), male sex (*P* < 0.0001), younger age [<4 years (*P* = 0.01)], prior use of antibiotics (*P* = 0.011), or history of UTI (*P* = 0.012) (Table 1) (28). Significantly higher rates of abnormalities, such as VUR, were found with non-*E. coli* UTIs (Table 1) (13, 26, 28). It is noteworthy that renal and bladder imaging methods are recommended for febrile UTIs caused by non-*E. coli* species (39). The percentages of *E. coli* (49%) and non-*E. coli* (51%) in patients with normal ultrasound were comparable (Table 1) (13).

#### 4.2.3. Association of UTIs with ESBL-producing and non-ESBL-producing uropathogens

One study of adults and children in Saudi Arabia found that significantly more UTI patients had non-ESBL-producing than ESBL-producing *E. coli*, and ESBL-producing *E. coli* were associated with VUR in children (*P* = 0.044) (Table 1) (12). The percentage of ESBL-producing *E. coli* was higher in male than female patients (*P* < 0.01); in neonates than older children and infants (*P* = 0.016) (34); and in recurrent than first UTIs (32). In contrast, no association was found between ESBL- or non-ESBL-producing bacteria and patient sex, age group, previous use of antibiotics, recurrent UTI, abnormal renal ultrasound, or abnormal VCUG in CA-UTI (Table 1) (22). Antibiotic use prior to hospital admission was recorded for 29.7% (28) and 40.6% (22) of patients (of which 27.2% had ESBL-producing bacteria) (22).

#### 4.2.4. Risk factors for recurrent or complicated UTIs

The prevalence of recurrent UTI was 28.5% (542/1,900) (Supplementary Table S4) (13, 15, 19, 20, 22, 24, 28, 29, 32). The rate of renal anomalies was higher in recurrent (50.7%) than single-episode UTI [10% (*P* < 0.005)] (13). In univariate and multivariate analyses, younger patient age (<6 years) and male sex, but not comorbidities (such as congenital abnormalities and immune disorders), were significantly associated with severe/complicated CA-UTI (defined as UTI which required hospitalization, presented with pyelonephritis, or recurrent UTI <30 days after initial diagnosis) (29).

## 5. Microbiology of pediatric UTIs in the GCC countries

### 5.1. Most commonly isolated pediatric urinary pathogens

*E. coli* was predominant (26.1%–100% of isolates), with a regional prevalence of 64.9% (1,898 *E. coli* among 2,925 uropathogens) (11, 13, 16–18, 22–28, 30, 32–34). ESBL-producing uropathogens were documented in 15 studies (Supplementary Table S4) (11, 12, 16–18, 22–26, 30, 32–34, 36). The regional prevalence of ESBL-producing *E. coli* and *K. pneumoniae* was 25.0% (480 of 1,918 *E. coli*) and 19.8% (37 of 187 *K. pneumoniae*), respectively (11, 12, 16–18, 22, 24–26, 30, 32–34). Half of all ESBL-producing uropathogens from CA-UTIs were isolated from children aged <1 year and more ESBL-producers were isolated in 2007–2009 than 2003–2006 (*P* = 0.05) (Table 1) (11).

*Klebsiella* spp. (predominantly *Klebsiella pneumoniae*) were the second most common uropathogens (3.8%–35.2% of isolates), with a regional prevalence for *K. pneumoniae* of 13.8% (377 isolates among 2,723 uropathogens) (11, 13, 17, 22–25, 27, 30).

Other isolated uropathogens included *Pseudomonas aeruginosa*, *Proteus mirabilis*, *Enterococcus* spp., and *Enterobacter* spp. (Supplementary Table S4) (11, 15, 17, 18, 22–27, 30, 32, 34, 37).

### 5.2. Antimicrobial resistance rates and phenotypes among isolates from pediatric UTIs

#### 5.2.1. Resistance rates to β-lactams among pediatric urinary isolates

Resistance among *E. coli* was highest to ampicillin (62.7%–82.8%) and cefazolin (94.0%) in Bahrain and Saudi Arabia (Table 2) (18, 34). Cefotaxime resistance was 28% in Kuwait (15) among *E. coli*, and cefuroxime resistance in Bahrain increased significantly from 2018 to 2020 (Table 1) (34). More than 90% of *E. coli* isolated from in-patients were susceptible to ertapenem and meropenem (27). In Oman, meropenem resistance was higher among non-*E. coli* than *E. coli* (including ESBL-producing isolates) (*P* < 0.001) for recurrent UTIs (32).

More than 96% of ESBL-producing *E. coli* from children in Bahrain (Table 2) (34) and all ESBL-producing isolates (83% *E. coli*) from Qatar were resistant to ceftriaxone (Table 1) (36).

Among all *K. pneumoniae*, >90% of isolates were susceptible to cefotaxime, ceftriaxone, and meropenem (Table 2) (11, 15, 16, 23, 27). Overall resistance to ampicillin (which is commonly used to treat UTI caused by *E. faecalis*) was 26.9% among enterococci (37).

#### 5.2.2. Resistance and susceptibility rates to antibiotic classes used to treat UTIs other than β-lactams

Resistance to trimethoprim-sulfamethoxazole among urinary *E. coli* was 36.8%–55.6% in Saudi Arabia (16, 18, 30, 32, 34), whereas low resistance (≤9%) or high susceptibility (>90%) was reported to amikacin, gentamicin, levofloxacin, nitrofurantoin or tigecycline in Bahrain, Kuwait, Oman, and Saudi Arabia (15, 16, 18, 27, 30, 34). In Bahrain, ciprofloxacin resistance in *E. coli* increased significantly from 2018 to 2020 (Table 1) (34). Colistin and nitrofurantoin resistance was higher in non-*E. coli* than *E. coli* (including ESBL-producing isolates) from recurrent UTIs (*P* < 0.001) (32).

No ESBL-producing isolates (*E. coli*, 83%) from Qatar were resistant to amikacin, with higher resistance to nitrofurantoin (13.0%), gentamicin (24.4%), and trimethoprim-sulfamethoxazole (59.7%) (Table 1) (36). Susceptibility among ESBL-producing *E. coli* from Bahrain was highest to nitrofurantoin and fosfomycin (71.0%–100%) (Table 2) (33). All carbapenem-resistant urinary Enterobacterales were resistant to ciprofloxacin, and susceptible to colistin and tigecycline (38).

Among *K. pneumoniae*, >90% of isolates were susceptible to the tested aminoglycosides (Table 2) (11, 15, 16, 23, 27). The susceptibility of *K. pneumoniae* to trimethoprim-sulfamethoxazole was 63.6% (27). Among enterococci from newborns, the lowest resistance was to ciprofloxacin (6.7%) (37).

#### 5.2.3. Multidrug resistance profiles among pediatric urinary isolates

Multidrug resistance [defined by the authors as resistance to ≥3 antimicrobial classes in the testing panel (Supplementary Table S3)], was found in 10.3% of *E. coli* (18). Another study found no significant association between age group, patient sex or congenital anomalies (including VUR) in pediatric UTI patients and urinary isolates exhibiting multidrug resistance (overall rate, 30.2%) (Table 1) (20). Their study determined a higher rate of multidrug resistance in recurrent vs. first episode UTIs (50.0% and 22.7%, respectively) (20).

### 5.3. Antimicrobial resistance genotypes

Most pediatric ESBL-producing *E. coli* isolates (61.1%) harbored the bla_CTX−M−G1_ gene, whereas 46.2% of ESBL-producing *K. pneumoniae* carried bla_SHV_, bla_TEM_ and bla_CTX−M−G1_ genes (Table 1) (36). The distribution of individual ESBL genes did not correlate with patient age (36).

Similar findings were demonstrated by a study in Qatar of pediatric ESBL-producing *E. coli* and *K. pneumoniae* (52.2% of isolates were urinary) (42). CTX-M group 1 enzymes (CTX-M-15, 87.8%) were the most prevalent ESBL enzymes and all isolates were resistant to ceftriaxone (42).

A study of carbapenem-resistant Enterobacterales (63.3% *E. coli*; 30% *K. pneumoniae*) in Qatar revealed that carbapenem resistance was largely attributed to a limited number of OXA-48-like and NDM carbapenemases, suggesting that certain international high-risk clones associated with other carbapenemase genes such as *bla*_KPC_-type, have not spread locally (38).

## 6. Antimicrobial management of pediatric UTIs in the GCC countries

### 6.1. Current antimicrobial treatment landscape in the GCC countries

For CA-UTI, SPIDS recommends first-line oral amoxicillin-clavulanic acid, cephalexin, cefuroxime, or cefprozil (39). The Qatar Ministry of Public Health (MOPH) recommends amoxicillin-clavulanic acid or a third-generation cephalosporin (3GC) as an empiric oral therapy, and gentamicin, amoxicillin-clavulanic acid, or ceftriaxone as empiric intravenous (IV) therapy (43). For infants and children hospitalized with a CA-UTI, an IV parenteral 3GC (such as ceftriaxone) is recommended (39). In-patients were mostly prescribed a 3GC (23), and patients hospitalized with a first UTI were given cefotaxime as empiric therapy before uropathogen susceptibilities were confirmed (15).

For in-patients with previous UTIs caused by ESBL-producing uropathogens, and in those who have been recently exposed to cephalosporins, gentamicin should be considered (39). Aminoglycoside therapy in combination with ceftriaxone may be indicated in critically ill patients and in those whose clinical condition worsens after starting the first-line antimicrobial therapy (39). A similar proportion of patients with ESBL-producing bacteria received IV antibiotics as in-patients or oral antibiotics as out-patients (27.8% and 26.1%, respectively) (22).

In the emergency department, most pediatric patients received a broad-spectrum antibiotic for CA-UTI (75.5%), mostly a cephalosporin (43.5%) or penicillin (44.2%), namely amoxicillin-clavulanic acid (29.4%) or cefprozil (23.0%) (Supplementary Table S4) (19). In a tertiary hospital, ceftriaxone (29.5%), vancomycin (25.0%), and meropenem (20.5%) were predominantly prescribed (27). In the in-patient and out-patient departments, nitrofurantoin (19%), trimethoprim-sulfamethoxazole (16%), and amoxicillin-clavulanic acid (15%) were mostly prescribed (Table 1) (30).

## 7. Gaps in the current knowledge on pediatric UTIs in the GCC countries

The evidence presented in this review indicates limited published data from the UAE and a lack of studies on HA-UTIs. Also, most studies specified a patient age of <14 years, while four studies included patients aged 14–18 years (12, 18, 19, 33). GCC data are lacking on predictors of treatment failure; clinical outcomes; epidemiological data on multidrug resistance; complicated UTI in addition to the implication of prophylaxis on resistance rates; and the correlation of antimicrobial resistance rates with previous antimicrobial use. Further studies are also warranted to monitor the trends in rates of resistance phenotypes and antimicrobial resistance over time.

## 8. Discussion

Fever was the predominant symptom of pediatric UTI in the GCC countries. UTIs were more prevalent in girls, overall, and in boys aged <5 years. The predominant uropathogen was *E. coli* (including ESBL-producing isolates). ESBL-producing uropathogens are an established cause of UTI in the region, whereas CRE organisms (predominantly *E. coli*, followed by *K. pneumoniae*) are emerging (38). Guidelines on pediatric UTI from the neighboring region of Asia also found a higher incidence of UTI in boys than girls of a younger age (≤3 months) (44).

Existing data suggest that ampicillin and trimethoprim-sulfamethoxazole are not recommended empiric agents for pediatric UTIs in the GCC region due to high resistance rates (18, 33, 34, 39). Furthermore, our review found that 3GCs are commonly prescribed in GCC countries for the empirical treatment of children with pyelonephritis that required hospitalization, despite high regional 3GC resistance (15, 39, 43). Of note is that 3GC resistance in our region is mainly attributed to CTX-M-type ESBL enzymes that exhibit higher *in vitro* activity against cefotaxime and ceftriaxone than other oxyimino-cephalosporins (36, 42). Nevertheless, observational studies have not observed significant differences in clinical response and outcome in ESBL UTI between patients started on 3GC and those initially receiving effective agents, and between UTI caused by resistant and susceptible strains in patients started on 3GC, suggesting that cefotaxime or ceftriaxone are reasonable empirical choices until antimicrobial susceptibility testing results are available (45–47).

Empirical use of aminoglycosides due to the high prevalence of uropathogens resistant to 3GC has been proposed in Kuwait (15). Gentamicin is recommended by SPIDS and Qatar MOPH, particularly in patients recently exposed to cephalosporins (39, 43). However, the prevalence of gentamicin resistance among ESBL-producing uropathogens, especially *E. coli*, is >20% in these countries (30, 36). Therefore, amikacin, which is increasingly used in many countries as a carbapenem-sparing option, may be a suitable option given the low amikacin resistance rates among ESBL-producing uropathogens in the GCC region, particularly with MIC ≤4 mg/L (15, 48–51)*.* Empirical treatment with carbapenems (meropenem if aged <3 months or ertapenem if aged >3 months) should be reserved for critically ill children, particularly those having a previous UTI caused by, or other risk factors for, ESBL-producing uropathogens. Alternatively, ceftazidime-avibactam, a novel cephalosporin/β-lactamase inhibitor combination that inactivates ESBL and OXA-48-type enzymes, could be considered a treatment option for ESBL pyelonephritis (52).

The treatment of UTIs caused by carbapenemase-producing uropathogens in our region is challenging. Although aztreonam is stable against NDM and 3GC against OXA-48 enzymes, none of these agents can be empirically used to treat UTI caused by NDM and OXA-48 producers as most isolates in our region co-produce CTX-M-type ESBL (38). It could be speculated that susceptibility to aztreonam might be restored by adding avibactam, making aztreonam-avibactam or ceftazidime-avibactam combined with aztreonam, theoretically appealing alternatives for pyelonephritis caused by NDM producers. Ceftazidime-avibactam and meropenem, for strains with meropenem MIC <8 mg/L, could be considered to treat pyelonephritis caused by OXA-48 producers (53, 54). Finally, oral nitrofurantoin or fosfomycin can be used to treat uncomplicated lower UTI caused by ESBL and carbapenemase-producing *E. coli* in our region.

In conclusion, our review showed that UTIs are increasingly caused by antimicrobial-resistant uropathogens in the pediatric population of GCC countries. Further epidemiological and clinical studies are needed to optimize diagnostic and antimicrobial stewardship strategies in pediatric UTIs in the GCC region.
