# Supplementary material for: Urinary tract infections in children from the Gulf Cooperation Council countries: a literature review (2011–2022)
Source: Front Pediatr. 2023 Jul 17;11:1163103. doi: 10.3389/fped.2023.1163103 (PMC10387756; doi:10.3389/fped.2023.1163103)
Supplement: Supplementary file 1 [file Table1.pdf]

Supplementary Table 1. Secondary search terms and related synonyms for literature on pediatric UTIs in the GCC countries (2011–2022)

Topic	Keywords/Synonyms
1. Gulf region	Gulf, Bahrain, Kuwait, Oman, Qatar, United Arab Emirates, UAE, Arab, Emirates, Middle East, Arabic, Arabian, KSA, Saudi Arabia, Gulf country, Gulf countries, Gulf state(s), GCC
2. Infections	Prevalence, incidence, infection rate, type(s) of infections (urinary tract infections, urine, UTI), patient demographics (hospital versus community), disease burden, hospital stay, mortality, clinical outcome, aetiology, etiology, epidemiology
3. Resistance	(Antimicrobial) resistance, surveillance, resistance rate(s), resistance mechanism(s), multidrug resistance, multi-drug resistance, multidrug-resistant, multi-drug resistant, MDR
4. Treatments	Treatment(s), antimicrobial(s), antibiotic(s), agent(s), medication(s), drug(s), treatment guideline(s), clinical outcome(s)
5. Pediatrics	18 years and below, female, male, patients unspecified
