# Supplementary material for: Urinary tract infections in children from the Gulf Cooperation Council countries: a literature review (2011–2022)
Source: Front Pediatr. 2023 Jul 17;11:1163103. doi: 10.3389/fped.2023.1163103 (PMC10387756; doi:10.3389/fped.2023.1163103)
Supplement: Supplementary file 2 [file Table2.pdf]

Supplementary Table 2. Details of the published studies on pediatric UTIs in the GCC countries (2011–2022)

Study reference / Center	Study type / Dates	Objectives	Inclusion criteria	Exclusion criteria	Method of urine collection	Statistical analysis
**Al-Saif et al., [11] (2012)** / King Abdulaziz Hospital, Al-Ahsa, Saudi Arabia	Retrospective / January 2003–April 2009	To assess susceptibility pattern of CA uropathogens in Saudi children	CA-UTI in non-hospitalized children	Children who had healthcare-associated UTI, recurrent UTI (defined as 2 proven episodes within 6 mo), neurogenic bladder, vesicostomy, and on intermittent catheterization. All possible contaminated urine samples were excluded including those who grew *Candida*, *Staphylococcus species*, or group B *Streptococcus*.	Urine bag, catheterization, midstream clean catch. No data on method of collection in 70% of pts.	Chi-square and Fisher exact test. A 2-sided *P*-value <0.05 was considered statistically significant.
**Al-Otaibi & Bukhari [12] (2013) /** King Khalid University Hospital,	Retrospective / June 2009–June 2011	To describe the epidemiology including risk factors of UTIs, and compare infections caused by ESBL-*E. coli* and non-ESBL strains	Hospitalized pts and out-patients (adult and pediatric pts) with culture-verified UTI	NA	NA	Chi-square test and Fisher's exact test. A statistically significant difference was considered when *P* <0.05.

Study reference / Center	Study type / Dates	Objectives	Inclusion criteria	Exclusion criteria	Method of urine collection	Statistical analysis
Riyadh, Saudi Arabia						
**Garout et al., [13] (2015) /** King Adbulaziz University Hospital, Jeddah, Saudi Arabia	Retrospective / October 2013–February 2014	To detect abnormal renal US findings in children aged <5 y with UTI, and compare the etiology of infection and abnormal US finding in cases with first episode of infection with those with recurrence of infection.	All children aged <5 y who had UTI confirmed by urine culture	Pts with colony count $<10^5$ CFU/mL, as well as those with bag urine collection	Transurethral catheterization (56.2%), midstream urine (42.5%), or suprapubic aspiration (1.3%). Main method in pts aged <2 y was transurethral catheterization (69.4%), and in those aged >2 y, midstream urine (78.6%).	Chi-square test. For all the statistical tests, a *P* <0.05 was considered statistically significant.
**Hendaus et al., [14] (2015) /** Hamad Medical Corporation, Doha, Qatar	Retrospective / January 2010–December 2012	To estimate prevalence of UTI in children hospitalized with bronchiolitis. To evaluate the effect of demographic and clinical characteristics and viral	Pts aged 0 to 24 mo hospitalized with acute bronchiolitis	All children with urinary tract abnormalities and those with rectal temperatures ≤38°C (100°F)	Only catheter urine samples were included to decrease the risk of contaminated urine	Univariate and multivariate logistic regression analyses. A two-sided *P* <0.05 was considered statistically significant.

Study reference / Center	Study type / Dates	Objectives	Inclusion criteria	Exclusion criteria	Method of urine collection	Statistical analysis
		etiology on the prevalence rates of UTI.				
**Husain et al., [15] (2015) /** Mubarak Al Kabeer Hospital, Kuwait	Retrospective / June 2011–May 2012	To evaluate the practicality of applying the new AAP guidelines (2011) for UTI in children aged 2-24 mo. To evaluate the value of the currently applied guidelines in detecting VUR and to the new guidelines will be safe and valuable. To identify if the currently empiric antibiotic used is suitable for the isolated bacteria.	Up to 12 y with both pyuria and urine culture of >50,000 CFU/mL of a single uropathogen in an appropriately collected specimen of urine	Mixed pathogens, no pyuria and <50,000 CFU/mL of bacteria	NA	Chi-square tests. A *P*-value of <0.05 was the cut-off for statistical significance.
**Sharef et al., [16] (2015) /** Sultan Qaboos University	Retrospective / September 2008–August 2012	To report common uropathogens and their antibiotic susceptibility in children with single episode UTI. To describe clinical presentation	≤14 y and had positive urine cultures	Pts with recurrent UTIs, neurological impairment, immune deficiencies and hematological malignancies. Pts with deficient data.	Mostly clean catch samples because the catheterization procedure is not widely	Chi-square test or Student's *t*-test and the Wilcoxon-Mann-Whitney U test. An a priori two-tailed level of significance was set at 0.05.

Study reference / Center	Study type / Dates	Objectives	Inclusion criteria	Exclusion criteria	Method of urine collection	Statistical analysis
Hospital, Muscat, Oman		and laboratory evaluation of these children.			accepted in our community	
**Kabbani et al., [17] (2016)** / King Abdulaziz Cardiac Center, Riyadh, Saudi Arabia	Retrospective / January 2012–December 2012	To determine the incidence, etiology, main risk factors, and outcome of UTIs in post-operative cardiac children admitted to the PCICU	All post-operative pts, aged <14 y, admitted to PCICU.	Pts admitted for other reasons.	Cultures collected in an aseptic technique by indwelling urinary catheters or suprapubic needle aspiration.	Poisson logistic regression, Wilcoxon Rank-Sum test, and Fisher's Exact Test. *P* values <0.05 were considered statistically significant.
**Alanazi et al., [18] (2018a)** / King Adbulaziz Medical City, Riyadh, Saudi Arabia	Retrospective / January 2008–March 2008	To determine the current prevalence and susceptibility to AMP, AMC, SXT, NIT, CFZ, and CIP amongst all *E. coli* isolated from pts with UTIs	Pts diagnosed with UTIs due to *E. coli*	NA	A properly collected midstream "clean catch" urine sample.	NA
**Alanazi [19] (2018b)** / King Abdulaziz Medical City,	Retrospective / First quarter of the year	To determine the prevalence of CA-UTIs among ED visits in Saudi Arabia, assess the pattern of antibiotic treatment of CA-UTIs,	All pts aged >6 mo admitted to the ED with	Incomplete antibiotic prescriptions, infants weighing <5 kg. Pts with acute complicated UTI, catheter-associated UTI, and	A properly collected midstream "clean catch" urine sample.	Pearson's chi-square test. For all statistical tests, *P* <0.05 was considered statistically significant. Prevalence of inappropriate antibiotic prescriptions

Study reference / Center	Study type / Dates	Objectives	Inclusion criteria	Exclusion criteria	Method of urine collection	Statistical analysis
Riyadh, Saudi Arabia	(January 2018–March 2018)	determine the prevalence and types of antibiotic-prescribing errors, and assess the cost of inappropriate antibiotic use in the treatment of CA-UTIs.	CA-UTIs during the first quarter of the year.	comorbidities (such as liver disease, renal insufficiency, malignant tumour, and AIDS).		was determined as the number of physician orders with one or more types of error divided by the total number of prescriptions and multiplied by 100. The prevalence of prescribing errors (selection, dose, frequency, and duration) was first calculated as mutually exclusive prevalence by dividing the number of errors over the number of antibiotic prescriptions multiplied by 100.
**Hisham et al., [20] (2018)** / King Abdulaziz University Hospital, Jeddah, Saudi Arabia	Retrospective / 2016–2017	To evaluate antimicrobial resistance patterns of UTI pathogens among children	Clinical diagnosis of UTI from the patient database of selected clinical settings.	Pts with any comorbidity and at stage of chronic condition.	NA	Correlation analysis was performed

Study reference / Center	Study type / Dates	Objectives	Inclusion criteria	Exclusion criteria	Method of urine collection	Statistical analysis
**Alfakeekh et al., [21] (2019)** / King Abdul-Aziz Medical City, Riyadh, Saudi Arabia	Retrospective, cross-sectional / January 2003–December 2013	To estimate the immunosuppressive burden, rate of infection and identify possible risk factors in PCNS requiring hospitalization	Children ≤ 14 years of age with the diagnosis of PCNS.	Diagnosis of congenital nephrotic syndrome, secondary nephrotic syndrome (e.g., IgA nephropathy, lupus nephritis), primary immunodeficiency, malignancy, pts on dialysis	NA	Mann-Whitney U test or Kruskal-Wallis test. A *P*-value of <0.05 was considered statistically significant difference.
**Awean et al., [22] (2019)** / Al Wakra Hospital, Hamad Medical Corporation, Qatar	Retrospective, cross-sectional / January 2016–December 2016	To estimate the prevalence of UTI due to ESBL bacteria in children. To identify possible risk factors for ESBL-UTI.	Pts aged 1 d–14 y, diagnosed to have UTI (a positive urine culture of single pathogen with >10^4 CFU/ mL) obtained by catheter, midstream urine sample, or any single colony for culture obtained by suprapubic aspiration.	UTI based on urine culture obtained by other means and culture result showing mixed growth.	Catheter, midstream urine sample, or by suprapubic aspiration.	Chi-square test and Fisher Exact or Yates corrected Chi-square tests; unpaired and Mann Whitney U tests; Pearson's correlation coefficients. Univariate and multivariate logistic regression. Two-tailed *P* values were considered statistically significant if <0.05.

Study reference / Center	Study type / Dates	Objectives	Inclusion criteria	Exclusion criteria	Method of urine collection	Statistical analysis
**Hameed et al., [23] (2019)** / King Abdulaziz Medical City, Riyadh, Saudi Arabia	Retrospective / 2006–2012	To investigate uropathogens and their resistance patterns in children presenting with their first admission for a UTI.	Age 0–14 years with a discharge diagnosis of UTI (as per AAP Clinical Practice Guidelines)	Age >14 y, HA-UTI, chronic renal failure, severe birth defects involving the urinary tract, immunodeficiency or immunosuppression and solid organ transplant	An appropriately collected urine specimen.	Independent samples t-tests. Significant differences were identified at P <0.05.
**Mohammed et al., [24] (2019)** / Salmaniya Medical Complex, Bahrain	Retrospective / June 2015–June 2017	To evaluate most common underlying organisms and associated urological anomalies in infants with UTIs.	All infants with UTIs (defined as the presence of 10,000 to 50,000 CFU/mL).	Urine samples collected by the bag, or urine sample showing mixed organisms indicating contaminations	Suprapubic aspiration or catheterization.	NA
**Abuzeyad et al., [25] (2020)** / King Hamad University Hospital, Bahrain	Retrospective / July 2015–July 2016	To evaluate the most common presentation and most common organism of UTI in different age groups. To evaluate appropriate empirical antibiotic therapy.	<14 y with a diagnosis of UTI, fever of unknown origin, urinary complaints, vomiting with or without fever and abdominal pain.	>14 y, structural urological anomalies, already diagnosed cases of UTI, and children with a long history of antibiotic therapy.	Fifty-four (51.9%) urine samples were collected by midstream clean catch method	NA

Study reference / Center	Study type / Dates	Objectives	Inclusion criteria	Exclusion criteria	Method of urine collection	Statistical analysis
			Pts were randomly selected.			
**Safdar et al., [26] (2020)** / King Abdulaziz University Hospital, Jeddah, Saudi Arabia	Retrospective /2015–2020	To determine the sensitivity and specificity of pyuria as a predictor of culture-proven UTI in the pediatric pts.	All pts aged ≤14 y who had UTI confirmed by culture.	Neonates with urogenital abnormalities or neonates admitted for surgical reasons. Asymptomatic pts, for whom a urine sample was taken for another purpose.	Midstream (59.1%), transurethral (37.4%), nephrostomy tube (2.6%), suprapubic aspiration, (0.9%)	Shapiro-Wilk test, Mann-Whitney U test, Pearson chi-square test, Fisher's exact test, or 2x2 tables.
**Alavudeen et al., [27] (2021)** / Tertiary care hospital, Abha, Saudi Arabia	Retrospective / October 2020–March 2021	To assess antibiotic of choice for empirical therapy and susceptibility pattern of uropathogens to commonly used antimicrobials	Relevant data related to microbiological isolates from confirmed cases of UTIs in pts aged <10 y.	Cultures with $<10^5$ CFU/mL and those already on antibiotic drugs. Non-bacterial uropathogens, incomplete antibiotic prescriptions, pts with acute complicated UTIs (e.g., acute pyelonephritis and UTIs with sepsis or bacteremia), catheter-associated UTI, and major	Urinary catheterization and midstream urine method as per the standard procedure.	t-test, ANOVA, and chi-square test. A *P* value <0.05 was considered statistically significant.

Study reference / Center	Study type / Dates	Objectives	Inclusion criteria	Exclusion criteria	Method of urine collection	Statistical analysis
				comorbidities (such as liver disease, renal insufficiency, malignant tumor, and AIDS)		
**Al Nafeesah et al., [28] (2022)** / King Abdulaziz Medical City, Riyadh, Saudi Arabia	Retrospective / 2006–2012	To compare demographic characteristics, radiological abnormalities, and other predictors of *E. coli* and non-*E. coli* UTIs in children in a tertiary care center	0–14 y, UTI defined as presence of both positive urinalysis (pyuria, bacteriuria) and positive urine culture result of ≥10,000 CFU/mL.	HA-UTI, underlying urogenital abnormalities, chronic renal failure, immunosuppression. Pts with positive urine cultures obtained from bag collection.	For younger children, catheterization or by suprapubic aspirate	Significant differences were identified at *P*-value <0.05
**Alrasheedy et al., [29] (2021)** / Ministry of Health tertiary hospitals, Saudi Arabia	Cross-sectional / April 2020–July 2020	To identify prevalence of UTI in children; risk factors for UTI including region in Saudi Arabia with highest prevalence; most common types of presentation; and risk factors for development of UTI.	Citizens or residents of Saudi Arabia, hospitalized for ≤2 d at time of diagnosis, age 1–10 y	HA-UTI.	NA	Univariate analysis using chi-square test and Mann-Whitney U test, multivariate analysis using binary logistic regression. *P* value was set at a significance level of <0.05. Severe/complicated UTI was defined as that which required hospitalisation, presented with

Study reference / Center	Study type / Dates	Objectives	Inclusion criteria	Exclusion criteria	Method of urine collection	Statistical analysis
						pyelonephritis, or recurrent UTI within 30 days after the initial diagnosis.
**Alzahrani et al., [30] (2021)** / King Fahad Hospital, Al Baha, Saudi Arabia	Retrospective / May 2017–April 2018	To determine the various bacteria causing UTI in pediatric pts and the antimicrobial resistance pattern	All pediatric pts aged 0–14 y with UTI.	Pts >14 y.	For infants, babies, and young children: clean catch method, bagged method and suprapubic bladder aspiration. For children aged >10 y, midstream urine or 20 mL urine was retrieved aseptically from the catheter after initial urine was voided.	NA
**Edun et al., [31] (2021)** / King Abdullah Specialized Children's	Retrospective, cross-sectional / November 2016–April 2017	To determine prevalence of UTI in children admitted for bronchiolitis. To establish whether routine urine investigations should continue	0–2 y old, admitted for bronchiolitis, with positive urinalysis and a positive culture.	Pts >2 y and if admitted to other departments such as Pediatric Cardiology.	Urine investigations via urinary catheterization are routinely done for pts with bronchiolitis.	Chi-square tests at a level of significance (*P* = 0.05).

Study reference / Center	Study type / Dates	Objectives	Inclusion criteria	Exclusion criteria	Method of urine collection	Statistical analysis
hospital, Riyadh, Saudi Arabia		to be carried out in children diagnosed with bronchiolitis.				
**El-Naggari et al., [32] (2021)** / Sultan Qaboos University Hospital, Muscat, Oman	Retrospective / September 2008–August 2012	To report most common uropathogens, and their antibiotic sensitivity pattern in children presented with first and recurrent UTI. To describe clinical presentation and laboratory evaluation in those children.	<14 y having a single or recurrent UTI	Neurological disability (laid up pts), immune deficiencies and hematological malignancies. Pts with deficient information accessible in electronic pts' records. Mixed cultures were rejected.	Either clean catch or catheter samples.	Ci-square test, where a *P*-value of 0.05 was set as cut-off of statistical significance.
**Saeed et al., [33] (2021)** / Salmaniya Medical Complex, Bahrain	Retrospective / January 2018–December 2019	To determine the antimicrobial susceptibility of ESBL-producing *E. coli* isolated from urinary samples to FOF and other antibiotics	All pure growth of *E. coli* with colony count ≥10^5 CFU/mL. Not limited to pediatric population.	Duplicate samples and mixed infections.	Midstream urine	Chi-squared test. A *P*-value of <0.050 was considered statistically significant.

Study reference / Center	Study type / Dates	Objectives	Inclusion criteria	Exclusion criteria	Method of urine collection	Statistical analysis
**Shaaban et al., [34] (2021)** / King Hamad University Hospital, Bahrain	Retrospective, cross-sectional / January 2018–May 2021	To describe the prevalence and the antimicrobial resistance patterns of the pathogens causing UTI in the pediatric age group	Pts aged < 14 y with confirmed UTI	Pts with chronic urinary tract conditions or neurodevelopmental problems involving the urinary tract.	NA	Chi-square tests. *P* ≤0.05 was considered statistically significant.
**Safdar et al., [35] (2015)** / King Abdulaziz University Hospital, Jeddah, Saudi Arabia	Cross-sectional / June 2014–August 2014	To assess the specificity and sensitivity of urinary NGAL in the early diagnosis of UTI	Febrile children with an average temperature of >38°C in the emergency or pediatric ward (0–14 y), clinically assessed and suspected to have UTI	NA	Catheter in infants and by midstream urine in older age groups	Shapiro-Wilk's test, Mann-Whitney U test, and Spearman correlation. The level of statistical significance was examined at *P* <0.05.
**Eltai et al., [36] (2018)** / Pediatric Emergency	Cross-sectional / February 2017–June 2017	To determine the phenotypic and genotypic profiles of antimicrobial-resistant Enterobacteriaceae among children with UTI	Children aged 0–15 y hospitalized with lower UTI as their primary diagnosis.	Samples that did not yield significant bacterial growth, those that had multiple organisms and samples with suspected contamination as per lab report.	Urinary catheter for all pts ≤2 y, cerebral palsy pts and pts under intermittent catheterization.	Non-parametric Kappa statistics, Pearson chi-square test, and Jacquard's coefficient. *P* value <0.05 was considered statistically significant.

Study reference / Center	Study type / Dates	Objectives	Inclusion criteria	Exclusion criteria	Method of urine collection	Statistical analysis
Center, Doha, Qatar				No duplicate samples were collected.	Otherwise, midstream urine.	
**Ahmad et al., [37] (2020)** / Children's Hospital, Riyadh, Saudi Arabia	Cross-sectional / September 2018–November 2018	To determine the prevalence and antibiotic sensitivity of UTI caused by *Enterococcus* in newborns	Newborn (age not defined)	Newborn UTI pts	Samples were obtained using sterile collection bags. Bags were removed as soon as urine was passed and the sample was transferred to a sterile container.	NA
**Al Mana et al., [38] (2021)** / Pediatric Emergency Center at Al-Saad, Hamad Medical Corporation, Qatar	Cross-sectional / October 2015–November 2019	To investigate genotypic profile of CREs among pediatric population with UTIs	Children presenting with lower UTIs as their primary diagnosis.	Samples that did not yield significant bacterial growth, those with multiple organisms, and samples with suspected contamination as per lab report. No duplicate samples were collected.	Urinary catheter for pts aged ≤2 y, cerebral palsy pts, and pts under intermittent catheterization. Otherwise, midstream catch.	NA

AAP, American Academy of Pediatrics; AIDS, acquired immunodeficiency syndrome; AMC, amoxicillin-clavulanic acid (Augmentin); AMP, ampicillin; ANOVA, analysis of variance; CA, community-acquired; CA-UTI, community-acquired urinary tract infection; CFU/mL, colony forming units per milliliter; CFZ, ; CIP, ; CRE, carbapenem-resistant Enterobacterales; d, days; ED, emergency department; ESBL, extended-spectrum β-lactamase; FOF, fosfomycin; HA-UTI, hospital-acquired UTI; IgA, Immunoglobulin A; mo, months; NA, data not available/reported; NGAL, neutrophil gelatinase-associated lipocalin; NIT, nitrofurantoin; PCICU, Pediatric Cardiac intensive care unit; PCNS, primary childhood nephrotic syndrome; pts, patients; SXT, trimethoprim-sulfamethoxazole (Bactrim or co-trimoxazole); US, ultrasound; UTI, urinary tract infection; VUR, vesicoureteral reflux; and y, years.
