# Supplementary material for: Urinary tract infections in children from the Gulf Cooperation Council countries: a literature review (2011–2022)
Source: Front Pediatr. 2023 Jul 17;11:1163103. doi: 10.3389/fped.2023.1163103 (PMC10387756; doi:10.3389/fped.2023.1163103)
Supplement: Supplementary file 3 [file Table3.pdf]

Supplementary Table 3. Antimicrobial susceptibility testing methodologies of the studies included in Table 2

**Study reference Country**	**Testing methodology**	**Panel of antimicrobial agents tested against *E. coli* and/or *K. pneumoniae***
**Al-Saif et al. [11] Saudi Arabia**	Antibiotics susceptibility testing was performed by Kirby-Bauer methods, confirmed, if required, by testing minimum inhibitory concentration using E. strip test. Interpretation followed the CLSI M100 guidelines. ESBL activity was detected by using E test ESBL CTX/CTX+clavulanic acid strip and CAZ/CAZ+clavulanic acid strip.<br>For a given uropathogen and a given antibiotic, antibiotics sensitivity as proportion rounded to the nearest whole number (the number of sensitive organisms/the total number of tested organisms) with its 95% CI calculated.	*E. coli* and *K. pneumoniae*: AMP, CXM, NIT, SXT, CTX, CAZ, CRO, GEN, AMK, TZP, and IPM.
**Hussain et al. [15] Kuwait**	N/A	*E. coli* and *K. pneumoniae*: AMP, AMC, AMK, CEF, CTX, CAZ, MEM, TZP, TMP, and NIT.
**Sharef et al. [16] Oman**	N/A	*E. coli* and *K. pneumoniae*: AMP, AMC, AMK, CIP, CTX, CRO, CXM, NIT, GEN, MEM, SXT, and TZP.
**Kabbani et al. [17] Saudi Arabia**	CLSI was used in our cases to determine the susceptibility of microbial organisms to antibiotics using the breakpoint dilution method.	AMP, AMK (only ESBL-*E. coli* and *K. pneumoniae*), CFZ (only *E. coli* and ESBL-*K. pneumoniae*), CTX, GEN, SXT, NIT, CIP, MEM (only ESBL-*E. coli*, *K. pneumoniae*, and ESBL-*K. pneumoniae*), and IPM.

Study reference Country	Testing methodology	Panel of antimicrobial agents tested against *E. coli* and/or *K. pneumoniae*
**Alanazi et al. 2018a [18] Saudi Arabia**	Sensitivity and resistance of *E. coli* isolates were determined using VITEK test method. Susceptibility interpreted according to the CLSI guidelines. Resistance of *E. coli* to one or more of three antibiotics (AMP, SXT, and CIP) were studied and classified to SSR (sensitive to AMP, sensitive SXT, and resistance CIP); RRR (resistance AMP, resistance SXT and resistance CIP); SSS (sensitive AMP, sensitive SXT and sensitive CIP); RRS (resistance AMP, resistance SXT and sensitive CIP); SRS (sensitive AMP, resistance SXT and sensitive CIP), and SRR (sensitive AMP, resistance SXT and resistance CIP). Multidrug resistance was defined as resistance to three or more antimicrobials.	Only *E. coli*: AMP, AMC, SXT, CIP, NIT, and CFZ.
**Hameed et al. [23] Saudi Arabia**	Resistance rates included isolates in the intermediate category.	*E. coli* and *K. pneumoniae*: AMP, AMC, CFZ, CTX, CIP, SXT, GEN, IPM, MEM, and NIT.
**Abyzeyad et al. [25] Bahrain**	N/A	Only *E. coli*: AMC and CXM.
**Alavudeen et al. [27] Saudi Arabia**	N/A	*E. coli* and *K. pneumoniae*: AMK, AMC, AMP, FEP, FOX, CRO, CXM, CIP, LVX, ETP, GEN, IPM, MEM, NIT, TZP, TGC, SXT, CTX, CAZ, and FOF.

Study reference Country	Testing methodology	Panel of antimicrobial agents tested against *E. coli* and/or *K. pneumoniae*
**Alzahrani et al. [30] Saudi Arabia**	The VITEK system was used to perform comprehensive confirmation and antibiotic susceptibility tests. The collected data were examined and interpreted in accordance with the CLSI's criteria.	*E. coli*: AMK, AMP, AMC, ATM, FEP, CTX, FOX, CAZ, CRO, CXM, CEF, CIP, CST, SXT, GEN, IPM, LVX, MEM, NAL, NIT, NOR, TZP, and TGC.<br>ESBL-*E. coli*: AMK, AMP, AMC, ATM, FEP, FOX, CAZ, CRO, CXM, CEF, CIP, SXT, GEN, IPM, LVX, MEM, NIT, NOR, and TZP.<br>*K. pneumoniae*: AMK, AMP, AMC, ATM, cedoxitine, FOX, CRO, CXM, CEF, CIP, SXT, GEN, IPM, LVX, MEM, NIT, NOR, TZP, and TOB.
**El-Naggari et al. [32] Oman**	Pure cultures of pathogenic organisms were tested for antimicrobial susceptibility based on CLSI guidelines.	Only *E. coli*: AMP, AMC, AMK, CIP, CTX, CRO, CXM, NIT, GEN, MEM, SXT, and TZP.
**Saeed et al. [33] Bahrain**	The antimicrobial susceptibility testing of all isolates was done by the standard Kirby-Bauer disk diffusion method using commercial disks (Oxoid) according to CLSI.	Only *E. coli*: CIP, SXT, NIT, and FOF.

Study reference Country	Testing methodology	Panel of antimicrobial agents tested against *E. coli* and/or *K. pneumoniae*
**Shaaban et al. [34] Bahrain**	Bruker MALDI-TOF was used for the identification of clinically relevant organisms and BD Phoenix M50 was used for both the identification of clinically relevant organisms as well as antibiotic sensitivity testing by measuring MIC.	*E. coli* and *K. pneumoniae*: AMP, NIT, SXT, CIP, AMC, CFZ, GEN, AMK, CXM, CRO, TZP, and CAZ.

AMC, amoxicillin-clavulanic acid (Augmentin or co-amoxiclav); AMK, amikacin; AMP, ampicillin; ATM, aztreonam; CAZ, ceftazidime; CEF, cephatholin; CFZ, cefazolin; CI, confidence interval; CLSI, Clinical and Laboratory Standards Institute; CRO, ceftriaxone; CST, colistin; CTX, cefotaxime; CXM, cefuroxime; ESBL, extended-spectrum β-lactamase; ETP, ertapenem; FEP, cefepime; FOF, fosfomycin; FOX, cefoxitin; GEN, gentamicin; IPM, imipenem; LVX, levofloxacin; MALDI-TOF, matrix-assisted laser desorption/ionization-time-of-flight; MEM, meropenem; MIC, minimum inhibitory concentration; NA, data not available/reported; NAL, nalidixic acid; NIT, nitrofurantoin; NOR, norfloxacin; SXT, trimethoprim-sulfamethoxazole (Bactrim or co-trimoxazole); TET, tetracycline; TGC, tigecycline; TMP, trimethoprim; TOB, tobramycin; and TZP, piperacillin-tazobactam (Tazocin).
