# Supplementary material for: Urinary tract infections in children from the Gulf Cooperation Council countries: a literature review (2011–2022)
Source: Front Pediatr. 2023 Jul 17;11:1163103. doi: 10.3389/fped.2023.1163103 (PMC10387756; doi:10.3389/fped.2023.1163103)
Supplement: Supplementary file 4 [file Table4.pdf]

Supplementary Table 4. Data from the studies on pediatric UTIs in the GCC countries (2011–2022)

**Study reference Country**	**Study cohort**	**Patient signs and symptoms**	**Abnormalities, comorbidities, risk factors, recurrence**	**Microbiology, antibiotic use**
**Al-Saif et al. [11] Saudi Arabia**	249 pts<br>Male: 62 (24.9%)<br>Female: 187 (75.1%)	NA	NA	258 isolates, most common: *E. coli*, 175 (67.8%), *Klebsiella*, 32 (12.4%).<br>10 (3.9%) isolates ESBL-positive (7, *E. coli*; 3, *Klebsiella*).<br>Our findings showed that sensitivity pattern of these uropathogens are better for CXM, NIT, CTX, and GEN than both AMP and SXT
**Al-Otaibi & Bukhari [12] Saudi Arabia**	339 pts<br>Adults: 106 (31.3%)<br>Pediatric (<18 y): 233 (68.7%)<br>Male: 96 (28.3%)<br>Female: 243 (71.7%)	Top three presenting symptoms for ESBL-*E. coli* versus non-ESBL-*E. coli*, (*P*-value of comparison):<br>Fever, 10 (8.9%) versus 24 (1.6%); (*P* = 0.609)<br>Dysuria, 2 (1.8%) versus 10 (4.4%); (*P* = 0.176)<br>Abdominal pain, 2 (1.8%) versus 7 (3.1%); (*P* = 0.375)	ESBL-*E. coli* versus non-ESBL-*E. coli* (*P*-value):<br>VUR: 5 (4.4) versus 2 (0.9) (*P* = 0.044)<br>Underlying comorbidities in 63 (19.0%) UTI episodes.<br>Risk factors for acquisition of ESBL *E. coli* included children with VUR.	113 caused by ESBL *E. coli*, 226 (176 pediatric pts, 50 adult pts) caused by non-ESBL-*E. coli*.<br>ESBL *E. coli* often resistant to antibiotics including 3rd generation cephalosporins, GEN and CIP (*P* <0.0001)

Study reference Country	Study cohort	Patient signs and symptoms	Abnormalities, comorbidities, risk factors, recurrence	Microbiology, antibiotic use
		Deaths, 10 (8.9%) versus 2 (0.9%); (*P* <0.0001)		
**Garout et al. [13] Saudi Arabia**	153 pts<br><2 y: 111 (72.5%)<br>>2 y: 42 (27.5%)<br>Male: 85 (55.6%)<br>Female: 68 (44.4%)	NA	Normal renal US: 100 (65.3%)<br>Abnormal renal US: 53 (34.6%)<br>Overall VUR, 29/48 (60.4)<br>Ninety percent of those with single UTI did not have anomalies.<br>Non-*E. coli* cases were associated with a higher percentage of abnormal renal US results (*P* = 0.006).	Single episode UTI: *E. coli* causative organism in 36/84 (42.9%) of pts and *K. pneumoniae* in 20.2%. Recurrent UTI: *E. coli* also most common organism (40.0%), followed by *K. pneumoniae* (19.0%)
**Hendaus et al. [14] Qatar**	835 pts hospitalized with acute bronchiolitis<br>Male: 510 (61.1%)<br>Female: 325 (38.9%)<br>Mean age: 3.47±2.99 mo	NA	Pts with urinary tract abnormalities excluded from study.<br>Being female (*P* =0.025) and older age at diagnosis (*P* = 0.006) were significantly associated with an increased risk for UTI.	50 of 518 pts tested had a positive urine culture (23 males, 27 females).<br>Antibiotic treatment was started for all children with positive urine cultures
**Husain et al. [15] Kuwait**	149 pts<br>Males: 79 (51.6%)	Top three presenting symptoms: Fever, 129 (86.6);	Overall VUR: 9/59 (15.3%); 6 of 46 with first UTI (13.0%); 3 of 13 with recurrent UTI, (23.1%).	Most common organisms: *E. coli* (103, 69.1%), *K. pneumoniae* (18, 12.1%, *P. aeruginosa* (8, 5.4%).

Study reference Country	Study cohort	Patient signs and symptoms	Abnormalities, comorbidities, risk factors, recurrence	Microbiology, antibiotic use
	Males, 0-6 mo: (31%)	Vomiting, 40 (26.8); Poor feeding, 29 (19.5)	Overall renal scarring: 5/50 (10.0%); first-episode, 3/5 and recurrent UTI, 2/5.	100% received empirical CTX, then switch to a narrower spectrum antibiotic after susceptibility testing.
**Sharef et al. [16] Oman**	175 pts<br>Female: 127 (72.6%)<br>Male: 48 (27.4%)<br>Age range: 2 wk–14 y<br>Median age: 4 y (IQR, 1–7)<br><1 y: 46 (26.3%)	Fever, 88 (50.3%)<br>Nausea and/or vomiting, 70 (40.0%)<br>Abdominal pain, 51 (28.6%)<br>Frequency, urgency, dysuria, 45 (25.7%)<br>Decreased activity, 21 (12.0%)<br>Reduced food intake/poor feeding, 20 (11.1%)	Pts with recurrent UTI were excluded from study.	Most common organisms: *E. coli* (120, 68.6%), *Klebsiella* spp. (30, 17.1%).<br>Resistance highest to AMP (75%) followed by SXT (47%) and AMC (43%). Resistance to IV antibiotics generally higher than oral.
**Kabbani et al. [17] Saudi Arabia**	413 post-operative cardiac pts (UTI in 29 pts)<br>Male: 16 (55.2%)<br>Female: 13 (44.8%)<br>Mean age 7.48 mo (SEM, 2.9)	Catheter-associated UTI definition: ≥1 of the following among fever (>38.0°C), hypothermia (<36.0°C), lethargy, vomiting, apnea, or bradycardia	Congenital anomalies of kidney and urinary tract: 9/29 (31.0%), mortality 2/29 (6.9%).<br>Congenital anomalies of kidney and urinary tract were seen more commonly in pts who developed UTIs (31%) compared to non-UTI cases (5% [$P < 0.05$]). Independent risk factors for developing UTIs post-cardiac surgery were the duration of urinary catheter placement ($P < 0.0001$), the	Most common organisms: *Klebsiella* (8, 27.6%), *Candida* (7, 24.1%), *E. coli* (6, 20.7%; 3 ESBL-positive *E. coli*).<br>The use of MEM was required in 7/10 cases with resistant organisms. In our surgical cases, we used first generation cephalosporins (CFZ) for 48 h as post-operative prophylaxis, mainly to minimize the risk of

Study reference Country	Study cohort	Patient signs and symptoms	Abnormalities, comorbidities, risk factors, recurrence	Microbiology, antibiotic use
			presence of urogenital abnormalities (*P* = 0.004), and the association with Down, DiGeorge or Noonan syndromes (*P* = 0.02).	surgical site infection. We frequently use prophylactically VAN and CAZ in neonates and patients with open sternums.
**Alanazi et al. [18] Saudi Arabia**	101 pts<br>Adults (18 to ≥65 y): 70 (69.3%)<br>Pediatric (<18 y): 31 (30.7%)	NA	NA	100% of tested isolates were *E. coli*.
**Alanazi [19] Saudi Arabia**	1449 pts<br>Adults (15 to ≥65 y): 1180 (81.4%)<br>Pediatric (<15y): 269 (18.6%)	NA	Pts with comorbidities were excluded from study.<br>Recurrent UTI in pediatric pts: 31 (11.5%).	Most common organism: *E. coli* (51%).<br>Broad-spectrum antibiotic prescriptions: 203 (75.5%)<br>Most common antibiotic classes: penicillin, 119 (44.2%), cephalosporin, 117 (43.5%)<br>Most common antibiotics: AMC, (29.4%), cefprozil (23%), CXM (16.4%), AMX (14.9%), SXT (5.8%), cephalexin (4.1%).
**Hisham et al. [20] Saudi Arabia**	63 pts<br>Male: 34 (54.0%)<br>Female: 29 (46.0%)<br><2 y: 32 (50.8%)	NA	Congenital abnormalities: 12 (19.0%)<br>Recurrent UTI: 18 (28.6%)<br>VUR: 6 (9.5%)	NA

Study reference Country	Study cohort	Patient signs and symptoms	Abnormalities, comorbidities, risk factors, recurrence	Microbiology, antibiotic use
	2 y: 22 (34.9%)<br>>2 y: 9 (14.3%)			
**Alfakeekh et al. [21]**<br>**Saudi Arabia**	111 pts with PCNS<br>84 PCNS pts with minor and major infections (76.4%)<br>25 pts with UTI (25.0%)<br><10 y: 74 (88.1%)<br>≥10 y: 10 (11.9%)<br>Male: 59 (70.2%)<br>Female: 25 (29.8%)	Main clinical symptoms: oedema (101, 91%), ascites (29, 26%)	NA	NA
**Awean et al. [22]**<br>**Qatar**	254 pts<br>Male: 58 (22.8%)<br>Female: 196 (77.2%)<br><1 y: 104 (40.9%)<br>1–3 y: 46 (18.1%)<br>3–6 y: 57 (22.4%)	NA	Abnormal US for 29 pts (11 with ESBL-bacteria; 18 with non-ESBL-bacteria)<br>VUR in 14 pts (6 with ESBL-bacteria; 8 with non-ESBL-bacteria)	68 (26.8%) had ESBL-bacteria; 186 (73%) had non-ESBL-bacteria.<br>Most common organisms: *E. coli*, 170 (66.9%; 55 ESBL *E. coli*); *Klebsiella* spp., 25 (9.8%; 8 ESBL-*Klebsiella* spp.).<br>Antibiotic use: 103 (40.6%); 28 (27.2%) with ESBL-bacteria and 75 (72.8%) with non-ESBL-bacteria.

Study reference Country	Study cohort	Patient signs and symptoms	Abnormalities, comorbidities, risk factors, recurrence	Microbiology, antibiotic use
	>6 y: 47 (18.5%)			
**Hameed et al. [23]** **Saudi Arabia**	202 pts<br>Male: 40 (19.8%; 27 [67.5%] were circumcised)<br>Female: 162 (80.2%<br><1y: 45 (22.3%)<br>1–14 y 157 (77.7%)	Fever: 170 (84.2%), Vomiting 104 (51.5%). Urinalysis results showed 92.6% of pts had urine white blood cell ≥5 cells per high power field and 92.1% had bacteriuria.	190 pts who had renal US 54% were abnormal with hydronephrosis and/or hydroureter as the most common abnormality). A cystourethrogram was performed in 96 pts and 69% had some degree of VUR.	Most common organisms: *E. coli*, 153 (75.7%), *K. pneumoniae*, 19 (9.4%), *P. aeruginosa*, 12 (5.9%).<br>16 (7.9%) organisms were ESBL-positive.<br>28 (35.4%) organisms were resistant to ≥3 antibiotics.
**Mohammed et al. [24]** **Bahrain**	125 pts<br>Male: 85 (68.0%)<br>Female: 40 (32.0%)<br>Median age: 29 d<br>Age range: 2–329 d	Fever, 74 (59.2%)<br>Neonatal jaundice, 46 (33.6%)	Renal US: 83 (66.4%)<br>Abnormal US finding: 29 (23.2%)<br>Hydronephrosis, 19 (15.2%)<br>VUR, 15 (12.0%)<br>Recurrent UTI: 15 (12.0%)	Most common organisms: *E. coli*, 69 (55.2%), *K. pneumoniae*, 44 (35.2%)<br>ESBL-producing organisms: 43.4% of the total *E. coli* and 31.8% of *K. pneumoniae* infections
**Abuzeyad et al. [25]** **Bahrain**	104 pts	Fever, 67 (64.4%)<br>Vomiting, 49 (47.1%)<br>Abdominal pain, 45 (43.3%)	Pts with urological anomalies and already diagnosed cases of UTI (recurrent UTI) were excluded from study	Most common organisms: *E. coli*, 36 (34.6%), ESBL-*E. coli*, followed by 31 (29.8%), *P. aeruginosa*, 5 (4.8%), *K. pneumoniae*, 4 (3.8%).<br>Among the empirical antibiotics used, CXM was found to be more sensitive than AMP among *E. coli*.

Study reference Country	Study cohort	Patient signs and symptoms	Abnormalities, comorbidities, risk factors, recurrence	Microbiology, antibiotic use
**Safdar et al. [26]** **Saudi Arabia**	315 pts (UTI in 115 pts)<br>Male: 56 (48.7%)<br>Female: 59 (51.3%)<br>Age at admission:<br>5.48±3.65 (0.33–14.00) y with *E. coli*<br>5.77±4.38 (0.08–17.00) y with non-*E. coli*	Pyuria: 80 (69.6%)	Pts with urological abnormalities were excluded from the study.<br>VUR: 17 (14.8%).<br>Pyuria was associated with a positive urine culture (*P* = 0.001). Blood white blood cell count, female gender, antibiotic use, and VUR also significantly predict UTI. Non-*E. coli* UTI strongly associated with VUR.	Most common organisms: *E. coli*, 38 (33.0%; ESBL-*E. coli*, 8 [26.7%]), *K. pneumoniae*, 25 (21.7%; ESBL-*K. pneumoniae*, 5 [25.0%]), *P. aeruginosa*, 15 (13.0%), *E. faecalis*, 12 (10.4%).
**Alavudeen et al. [27]** **Saudi Arabia**	132 pts<br>Male: 75 (56.8%)<br>Female: 57 (43.2%)<br>0–2 mo: 36 (13.6%)<br>2–12 mo: 57 (43.2%)<br>>12–48 mo: 15 (11.4%)<br>>48 mo: 24 (18.2%)	Top three presenting symptoms:<br>Fever, 111 (84.1%), diarrhea, 24 (18.2%), vomiting, 9 (6.8%)	Bladder mass: 3 (2.3%), bilateral hydronephrosis. 3 (2.3%), hydronephrosis, 3 (2.3%), VUR, 2 (2.3%)	Most common organism: *E. coli*, 42 (31.8%), *K. pneumoniae*, 33 (25.0%), *P. aeruginosa*, 9 (6.8%), *E. faecium*, 9 (6.8%).<br>Most common antibiotics: CRO (39, 29.5%), VAN (25.0%), MEM (20.5%), CXM (15.9%). Cephalosporins were the most commonly prescribed class.<br>Almost 99% of pts were administered drugs intravenously.

Study reference Country	Study cohort	Patient signs and symptoms	Abnormalities, comorbidities, risk factors, recurrence	Microbiology, antibiotic use
**Al Nafeesah et al. [28] Saudi Arabia**	202 pts<br>Mean age ± SD:<br>5.2 ± 0.28 y with *E. coli*<br>3.72 ± 0.48 y with non-*E. coli*	NA	Abnormal renal US, 100 (49.5%; 70 [48.3%] with *E. coli* and 30 [66.7%] with non-*E. coli*)<br>Abnormal VCUG: 96 (47.5%; 63 [51.7%] with *E. coli* and 33 [76.7%] with non-*E. coli*)<br>Past history of UTI: 80: (39.6%; 53 [34.6%] with *E. coli* and 27 [55.1%] with non-*E. coli*).<br>Non-*E. coli* UTIs were much more common in male subjects (*P* <0.0001) and younger children (*P* = 0.01). There was also a significant association with prior use of an antibiotic and previous history of UTI (*P* = 0.011 and 0.012, respectively). In children with UTIs caused by non-*E. coli* pathogens, abnormalities in renal US and VCUG were seen in 66.7% and 76.7% of patients, respectively, both significantly higher than in *E. coli* UTIs (*P* = 0.008 and *P* = 0.01, respectively). A higher frequency of children with *E. coli* UTIs had a length of stay of ≤1 week than non-*E. coli* UTIs.	*E. coli*, 153 (75.7%) and non-*E. coli*, 49 (24.3%; most commonly *K. pneumoniae*, followed by *P. aeruginosa*))<br>Received antibiotics prior to admission: 60 (29.7%; 38 [24.8%] with *E. coli* and 22 [44.9%] with non-*E. coli*)
**Alrasheedy et al. [29] Saudi Arabia**	280 pts with UTI<br>Male: 118 (42.1%)<br>Female: 162 (57.9%)<br>1–3y: 64 (22.9%)	Most common presenting symptom was urethritis, 131 (46.8%)	Most common comorbidities: vitamin deficiency, 24 (8.5%); diabetes mellitus, 16 (5.8%); hydronephrosis, 7 (2.2%).	NA

Study reference Country	Study cohort	Patient signs and symptoms	Abnormalities, comorbidities, risk factors, recurrence	Microbiology, antibiotic use
	3.1–6y: 101 (36.1%)<br>6.1–8y: 27 (9.6%)<br>8.1–10y: 57 (20.4%)<br>Unknown: 30 (10.7%)<br>Mean age with UTI: 4.5–5 y			
**Alzahrani et al. [30]**<br>**Saudi Arabia**	118 pts<br>Male: 37 (31.4%)<br>Female: 81 (68.6%)<br>0–1 mo: 2 (1.7%)<br>1 mo–1 yr: 23 (19.5%)<br>1–3 y: 18 (15.3%)<br>3–6 y: 33 (28.0%)<br>6–12 y: 29 (24.6%)	NA	NA	118 isolates, most commonly: *E. coli* (44.1%), ESBL-*E. coli* (11.9%), *K. pneumoniae* (9.3%), *E. faecalis* (7.6%). Most commonly prescribed antibiotics: NIT (19%), SXT (16%), AMC (15%), CXM (10%)

Study reference Country	Study cohort	Patient signs and symptoms	Abnormalities, comorbidities, risk factors, recurrence	Microbiology, antibiotic use
	12–14 y: 13 (11.0%)			
**Edun et al. [31] Saudi Arabia**	407 pts<br>Urine investigation in 270 (66.3%) pts	NA	NA	NA
**El-Naggari et al. [32] Oman**	405 positive urine cultures<br>175 pts (single episode)<br>Age range: 2 wk–14 y<br>Median age: 4 y (IQR, 1–7 y)<br>Male: 93 (23.0%)<br>Female: 312 (77.0%)<br>74 pts (recurrent UTI)	Top presenting symptoms for first-episode versus recurrent UTI (*P*-value of comparison):<br>Fever, 88 (50.3%) versus 57 (24.8%); (*P* <0.001)<br>Nausea and/or vomiting, 70 (40.0%) versus 25 (10.9%); (*P* <0.001)<br>Nonspecific abdominal pain, 51 (28.6%) versus 31 (13.5%); (*P* <0.001).<br>Frequency, urgency, dysuria, 45 (25.7%) versus 57 (24.8%), (*P* = 0.775)	Recurrent UTI:<br>230/405 (56.8%)	Single episode most common organisms:<br>*E. coli*, 307 (75.8%); *K. pneumoniae*, 42 (10.4%); *Enterococcus*, 14 (3.5%).<br>Recurrent UTI most common organisms:<br>*E. coli*, 120 (68.6%); *K. pneumoniae*, 30 (17.1%); *Enterococcus*, 7 (4.0%).

Study reference Country	Study cohort	Patient signs and symptoms	Abnormalities, comorbidities, risk factors, recurrence	Microbiology, antibiotic use
	Age range: 2 wk–14 y<br>Median age: 7 y (IQR, 4–9y).<br>Male: 47 (26.9%)<br>Female: 128 (73.1%)			
**Saeed et al. [33]**<br>**Bahrain**	3044 pts<br>Adults (16 to >50 y): 2440 (80.2%)<br>Pediatric (≤15 y): 604 (19.8%); <1 y, 248 (8.1%); 1–15 y: 356 (11.7%)	NA	NA	100% of tested pediatric isolates were *E. coli*: 216/604 (35.8%) were ESBL-*E. coli*; 5/604 (0.8%) were CR-*E. coli*. FOF is an ideal oral antibiotic and more suitable than the other three antibiotics included in our study.
**Shaaban et al. [34]**<br>**Bahrain**	242 pts	NA	NA	Most common organisms: *E. coli* (166, 68.6%; [29, 23.5% were ESBL-producing]), *K. pneumoniae* (25, 10.3%), *P. mirabilis* (12, 5.0%), *P. aeruginosa* (8, 3.3%).

Study reference Country	Study cohort	Patient signs and symptoms	Abnormalities, comorbidities, risk factors, recurrence	Microbiology, antibiotic use
**Safdar et al. [35] Saudi Arabia**	73 pts (UTI in 31 pts)<br>Male: 33 (45.2%)<br>Female: 40 (54.8%)	NA	NA	NA
**Eltai et al. [36] Qatar**	727 pts<br>201 ESBL-producing isolates<br>Male: 34 (16.9%)<br>Female: 167 (83.1%)<br><2 y: 60 (29.9%)<br>2–5 y: 82 (40.8%)<br>6–15 y: 59 (29.4%)	Mainly fever and dysuria	NA	635 Enterobacterales isolates (201 ESBL-producing, 31.7%). Most common ESBL-producing organisms: *E. coli*, 166 (82.6%), *K. pneumoniae*, 22 (10.9%).
**Ahmad et al. [37] Saudi Arabia**	317 urine samples from pts<br>30 (9.5%) Enterococcal strains<br>Male: 17 (56.6%)	NA	NA	100% of tested isolates were *Enterococcus* spp.<br>100% of *Enterococcus* spp. were resistant to CLI but IPM and AMC could be used for the treatment of newborn UTIs caused by enterococcal strains.

Study reference Country	Study cohort	Patient signs and symptoms	Abnormalities, comorbidities, risk factors, recurrence	Microbiology, antibiotic use
	Female: 13 (43.4%)			
**Al Mana et al. [38] Qatar**	29 pts<br>Male: 8 (27.6%)<br>Female: 21 (72.4%)<br><2 y: 5 (17.2%)<br>2–5 y: 13 (44.8%)<br>6–15 y: 11 (37.9%)	Mainly fever and dysuria	NA	30 CRE isolates. Most common CR organisms: *E. coli*, 19 (63.3%), *K. pneumoniae*, 9 (30.0%).<br>Most common carbapenemases: OXA-48-like enzymes (46.6%) and NDM enzymes (40%).

AMC, amoxicillin-clavulanic acid (Augmentin); AMP, ampicillin; AMX, amoxicillin; CFZ, cefazolin; CR, carbapenem-resistant; CR, carbapenem-resistant Enterobacterales; CRO, ceftriaxone; CTX, cefotaxime; CXM, cefuroxime; d, days; ESBL, extended-spectrum β-lactamase; FOF, fosfomycin; GEN, gentamicin; IPM, imipenem; IQR, interquartile range; MEM, meropenem; mo, months; NA, data not available/reported; NIT, nitrofurantoin; PCNS, primary childhood nephrotic syndrome; pts, patients; SD, standard deviation; SEM, standard error of the mean; SXT, trimethoprim-sulfamethoxazole (Bactrim or co-trimoxazole); US, ultrasound; UTI, urinary tract infection; VAN, vancomycin; VCUG, voiding cystourethrogram; VUR, vesicoureteral reflux; wk, weeks; and y, years.
